# Cancer and treatment specific incidence rates of immune-related adverse events induced by immune checkpoint inhibitors: a systematic review

**DOI:** 10.1038/s41416-024-02887-1

**Published:** 2024-11-03

**Authors:** Bishma Jayathilaka, Farah Mian, Fanny Franchini, George Au-Yeung, Maarten IJzerman

**Affiliations:** 1 Pharmacy Department, Peter MacCallum Cancer Centre, Melbourne, VIC Australia; 2 Sir Peter MacCallum Department of Oncology, Faculty of Medicine, Dentistry and Health Sciences, University of Melbourne, Melbourne, VIC Australia; 3 Cancer Health Services Research Unit, Centre for Cancer Research, Faculty of Medicine, Dentistry and Health Sciences, University of Melbourne, Melbourne, VIC Australia; 4 Department of Medical Oncology, Peter MacCallum Cancer Centre, Melbourne, VIC Australia; 5 Erasmus School of Health Policy & Management, Erasmus University, Rotterdam, The Netherlands

**Keywords:** Cancer immunotherapy, Adverse effects, Epidemiology

## Abstract

**Background:**

Immune-related adverse events (irAE) induced by immune checkpoint inhibitors (ICI) are a treatment-limiting barrier. There are few large-scale studies that estimate irAE prevalence. This paper presents a systematic review that reports the prevalence of irAE by cancer type and ICI.

**Methods:**

A systematic review was undertaken in MEDLINE OVID, EMBASE and Web of Science databases from 2017–2021. A total of 293 studies were identified for analysis and, of these, event rate was calculated for 272 studies, which involved 58,291 patients with irAE among 305,879 total patients on ICI. Event rate was calculated by irAE and ICI type.

**Results:**

Mean event rate for general irAE occurrence across any grade was 40.0% (37.3–42.7%) and high grade was 19.7% (15.8–23.7%). Mean event rates for six specific types of irAE are reported. Mean event rate for ICI monotherapy was 30.5% (28.1–32.9%), 45.7% (29.6–61.7%) for ICI combination therapy, and 30.0% (25.3–34.6%) for both ICI monotherapy and combination therapy.

**Conclusion:**

This systematic review characterises irAE prevalence across current research that examines irAE risk factors across cancers and ICI. The findings confirms that irAE occurrence is very common in the real-world setting, both high grade and irAE across any grade.

## Background

Immune checkpoint inhibitor (ICI) therapies have transformed the cancer treatment landscape by enabling the immune system to identify and eliminate tumour cells. The blockade of inhibitory immune pathways to promote T-cells significantly enhances the potential for anti-tumour immune response in the presence of cancer [1–3]. Cytotoxic T-lymphocyte associated protein 4 (CTLA-4), programmed cell death protein 1 (PD-1) and programmed cell death ligand 1 (PD-L1) are the most widely studied checkpoint pathways and have been the focus of checkpoint inhibitor development over the past two decades [1, 2, 4]. Since the first approvals, the use of ICI has expanded from advanced melanoma and non-small cell lung cancer (NSCLC) to various cancer types and in earlier stages of disease [5]. However, ICI are associated with immune-related adverse events (irAE), which are a unique range of inflammatory complications and are thought to result from reactivated cellular immunity [6–8]. Despite the significant challenge that irAE pose to the use of ICI as cancer treatment, the risks or contributors of irAE are poorly understood.

The understanding of mechanisms that underpin the development of irAE are still evolving, making it difficult to precisely define risk factors that predispose individuals to develop these adverse events [9, 10]. Nonetheless, emerging observations from real-world data provide some insights into potential novel factors that may contribute to irAE [11]. Firstly, it is recognised that the immune system is the driver of irAE and, logically, other conditions that are caused by immune dysregulation may amplify or influence development of irAE. Pre-existing autoimmune diseases, such as rheumatoid arthritis and inflammatory bowel disease, are proposed risk factors for irAE [12]. Autoimmune diseases may be associated with irAE diagnosis and autoimmune flare may occur during ICI treatment [13]. Secondly, genetic differences may influence an individual’s susceptibility to irAE [14]. For example, several single nucleotide polymorphisms and variations in gene expression have found to be associated with irAE in specific cancer types [14–16]. Thirdly, with the expanding use of ICI in different patient populations and cancers, there appears to be an association between cancer type and occurrence of different types of irAE [17]. Additionally, baseline factors that are recognised to contribute to medication-related toxicity are relevant to consider for development of irAE. Factors such as performance status, co-morbidities, concurrent medications, organ function and tolerance to previous treatment are generally reviewed prior to commencing ICI treatment.

The most well-studied factors that lead to the development of irAE are type and dosage of ICI. Specific ICI agents exhibit different toxicity patterns. As monotherapy, CTLA-4 inhibitors are considered to pose the highest overall risk of irAE compared to PD-1 and PD-L1 inhibitors [8, 18]. This is attributed to activity of CTLA-4 inhibitors in secondary lymphoid tissue while PD-1 and PD-L1 inhibitors are thought to active in the tumour microenvironment [19]. Consequently, irAE are more prevalent with combination anti-CTLA-4 and anti- PD-1/L1 therapy [18, 19]. In melanoma, the overall incidence of high grade irAE with combination therapy compared to anti-PD-1 monotherapy is approximately 55–60% versus 10–20% [20, 21].

The occurrence of irAE vary by affected organ, time of onset and severity. Gastrointestinal, endocrine, and dermatologic irAE occur frequently and can present at varying degrees of severity. Neurologic, cardiac, and pulmonary toxicities can result in fatal irAE. Fatal irAE are less common affecting approximately 0.3–1.3% of treated patients and generally occur in the early course of treatment [22]. The occurrence of irAE can vary across different cancer types. Gastrointestinal and dermatologic irAE may occur more commonly in melanoma compared to NSCLC while higher occurrence of dermatitis, arthritis and myalgia may appear in NSCLC compared to melanoma [18].

As toxicity data reported from previous studies comes from different sources (e.g. randomised controlled trials, registries, case series), the reported incidence of irAE vary widely. Data from registries may not be representative of all ICI patient populations or under-report low grade toxicities that are routinely managed in the oncology setting. To further improve clinical management as well as to quantify the impact of irAE there is a need to obtain more accurate estimates. This is also imperative for epidemiological studies, where accurately quantifying the relationship between risk factors and irAE occurrences hinges on reliable incidence reporting.

In the absence of comprehensive studies estimating the occurrence of irAE, we have undertaken a systematic approach to identify original research investigating irAE induced by CTLA-4, PD-1 and PD-L1 inhibitors at any phase of cancer treatment, among current literature that examines irAE risk factors or predictors. This paper delivers a systematic review that pinpoints the prevalence of irAE and sheds light on the characteristics of current literature regarding irAE risk factors and predictors. Our analysis outlines irAE occurrences by cancer type, stage, and ICI treatment, accounting for study design, analysis method, and study population as potential bias sources in these estimates.

## Methods

### Data sources and search strategy

A systematic search was conducted following the Preferred Reporting Items for Systematic Reviews and Meta-analysis (PRISMA) Statement [23]. The protocol for this study was registered with PROSPERO in March 2022 (CRD42022310127). Systematic literature search was conducted in MEDLINE OVID, EMBASE and Web of Science databases for the period January 2017 to December 2021, inclusive, with English language limit applied (based on language proficiency of the reviewers). The search strategy is provided in Supplementary Table S1. The search was conducted using the following PICOS format: P – Adult patients with cancer with immune-related adverse event/s; I – receiving immune checkpoint inhibitor treatment; C – no immune-related adverse event/s; O – differences in population characteristics across treatments and cancers; S – original research (including case series, case-control studies, observational studies, cohort studies, pharmacovigilance studies, randomised controlled trials, systematic reviews, and meta-analyses).

Studies identified in databases were imported into COVIDENCE where two researchers independently conducted screening of title and abstract to remove ineligible studies. Where studies potentially met the eligibility criteria, full texts were retrieved and screened by two researchers. Disagreements about eligibility for inclusion were resolved by consensus discussion between two researchers (Jayathilaka and Mian). Additionally, references in included studies were manually searched. Only full texts and supplementary materials available via institutional library database access proceeded to data extraction.

### Eligibility criteria

A broad approach was undertaken for this review encompassing any cancer diagnosis and all irAE of any grade. We included only original research in peer-reviewed scientific journals. Inclusion criteria was limited to 1) human studies; 2) involving adult participants with cancer; 3) immune checkpoint inhibitor treatment with either anti-CTLA-4, anti-PD-1, anti-PD-L1 or combination between any of these treatments; and 4) contained irAE occurrence, incidence, or reporting. Studies that contained individual patient level or population data on irAE risk factors, predictors or association were identified, including those with a positive or negative for correlation to irAE and where a correlation could not be established. Studies describing irAE management or treatment, prognostic risk or predictors, treatment efficacy or outcome predictors, or imaging-guided detection or diagnosis of irAE were included if there was patient, clinical or laboratory/biological data about irAE in article full text or its supplementary materials. Comparative studies involving immune checkpoint inhibitors versus any other cancer therapy (such as chemotherapy) were included if data about descriptors or risk of irAE was reported separately. Studies were excluded for any of the following criteria: 1) non-English language; 2) animal studies; 3) case reports or individual case studies; 4) meeting abstracts, commentary or letters to the editor; 5) immunotherapy other than immune checkpoint inhibitors specified in inclusion criteria; 6) non-cancer indications; 7) treatment-related adverse event or autoimmune disease that is not considered an irAE or not induced by immune checkpoint inhibitor treatment; 8) immune checkpoint inhibitor therapy in combination with another cancer treatment modality (such as chemotherapy, radiotherapy, molecular targeted therapy). Studies that reported exclusively on response, prognosis or efficacy to immunotherapy or any other cancer treatment, imaging-guided detection of response, or cost evaluation or effectiveness were excluded.

### Data extraction and quality assessment

All data extraction was completed in COVIDENCE. One researcher conducted data extraction for all included studies. Due to the volume of included studies, independent data extraction was completed for a random selection (approximately 20%) of studies by a second researcher with reference to a standardised extraction template. The following details were extracted: author, publication year, country/region, study design, source of patient population and/or data, aims, study period, total sample size, number of patients on immune checkpoint inhibitor treatment, number of patients with general and/or specific irAE, type of cancer and stage (if specified), name or type of ICI, details of all irAE reported and grade (if specified), definition of irAE, patient demographic details (including age, gender, ethnicity, weight, body mass index, smoking status, performance score), and details on proposed or established irAE risk factors or predictors. The latter involved collection of granular details relating to risk factors and irAE including patient, clinical, laboratory or imaging details, result, and method of statistical analysis to assess association (if performed), and stage of detection or measurement of risk factor or predictor. Quality and generalisability of studies were not assessed with the intention that this descriptive summary will highlight the variance in study design and level of evidence available in current literature.

### Data synthesis and analysis

Three population characteristics were selected to include in this review: cancer type, cancer stage, and specific ICI treatments. Data were summarised using descriptive statistics with event rate calculated to present the proportion of patients with irAE among the study population on ICI, where reported. Event rate was calculated for studies where number of patients with irAE among study population who received ICI were reported. This was calculated by irAE type and treatment type.

## Results

**Fig. 1:**
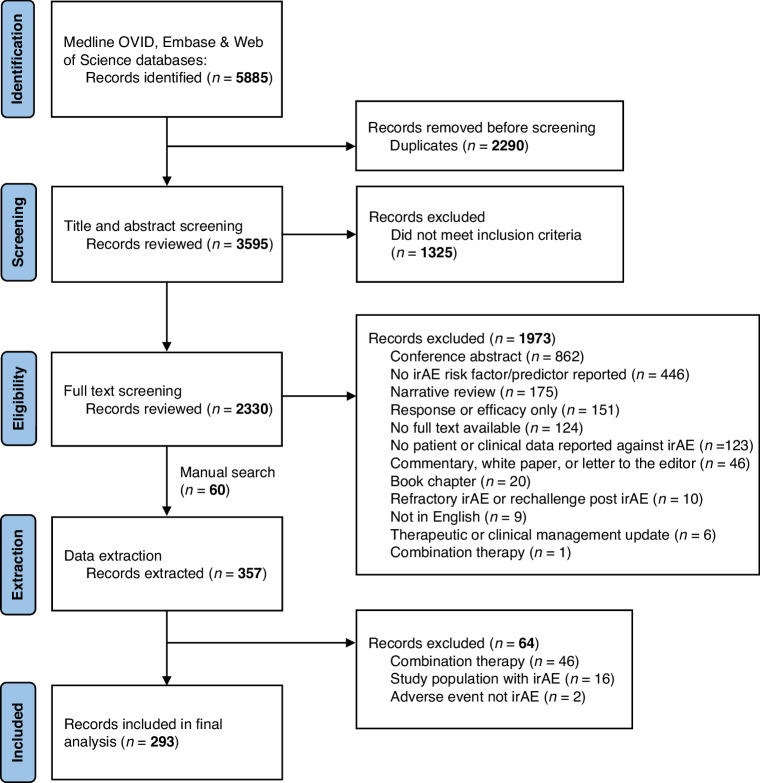
PRIMSA flowchart.

The PRISMA flowchart is shown in Fig. 1 for identification, screening, and selection of study records, including reason for exclusion.  A total of 5885 studies were identified in the three databases. After excluding duplicates, 3595 studies remained. After reviewing title and abstract screening and manual search of references, 2230 studies met the PICOS criteria and were eligible for full text screening. Following this, 357 studies proceeded to data extraction where additional studies were excluded. Finally, 293 studies were included for analysis. Details of search strategy are available in Supplementary Table S1.

### Characteristics of included studies

Study design and total patient numbers of the included studies are shown in Tables 1 and 2. In 293 included studies, irAE were reported in 58,291 patients among 305,879 total patients who were reported to have received ICI treatment. There were 24 studies that did not report on the number of patients with irAE and/or the study population size. There were 221 retrospective studies including 187 cohort or observational, 14 case-control, nine pharmacovigilance or surveillance, eight clinical trial subsets and three experimental/exploratory/pilot studies. Retrospective studies accounted for the majority of ICI patients included: 240,683 patients (78.7%). There were 55 prospective studies including 31 cohort or observational, 18 experimental/exploratory/pilot studies and six clinical trial subsets accounting for 4462 patients (1.5%). Additionally, there were 17 systematic reviews or meta-analyses containing 60,734 patients (19.9%). Full details of study characteristics are shown in Supplementary Table S2.

**Table 1:** Overview of different study designs of included studies. *n* = *293 studies*.

Study design	Retrospective	Prospective
Cohort or observational	187 (63.8%)	31 (10.6%)
Clinical trial or subset analysis	8 (2.7%)	6 (2.0%)
Experimental, exploratory, or pilot	3 (1.0%)	18 (6.1%)
Case control or matched case control	14 (4.8%)	–
Pharmacovigilance or surveillance	9 (3.1%)	–
Systematic review and/or meta-analysis	17 (5.8%)	–

**Table 2:** Summary of study population and number of patients with irAE reported in included studies. *n* = *293 studies*.

Study design	ICI study population	Number of patients with irAE
Cohort or observational	129,502^*^	16,365^†^
Clinical trial or subset analysis	9783	2496
Experimental, exploratory, or pilot	27,400	4367^‡^
Case control or matched case control	23,693^§^	3323
Pharmacovigilance or surveillance	54,767^‖^	23,705^¶^
Systematic review and/or meta-analysis	60,734^#^^§^	8035^∆^

For all study designs, study population is defined as patients who received ICI. In total, 24 studies had insufficient reporting on number of patients with irAE and/or study population on ICI: ^*^not reported in two studies; ^†^not reported in five studies; ^‡^not reported in one study; ^§^not reported in one study; ^‖﻿^not reported in seven studies; ^¶^not reported in three studies; ^#^not reported in one study; ^∆^not reported in eight studies.

### Cancer type

Of all the included studies, 170 (58.0%) performed a cancer-specific approach and 123 (42.0%) performed a pan-cancer approach to data analysis. Among all studies that performed a cancer specific approach, 81 focused on lung cancer (74 NSCLC, three NSCLC or SCLC, four other lung cancers), 62 studies focused on skin cancer (61 melanoma, one other skin cancers), three on renal cell carcinoma, two studies each on gastric cancer, head and neck cancer and urothelial carcinoma, one study each on liver, upper gastrointestinal cancer, Hodgkin’s lymphoma, and fifteen studies that included multiple specific cancer types.

### Treatment type

Among all studies, 193 (65.9%) focused on monotherapy across any class of ICI, 92 (31.4%) studies included both monotherapy and combination ICI therapies while eight (2.7%) focused solely on combination therapies.

### Immune-related adverse events

Among all studies, 162 (55.3%) reported on general irAE occurrence, 124 (42.3%) reported on specific irAE type(s), while seven studies (2.3%) reported on both specific irAE type(s) and general irAE occurrence. Overall, 46 (15.5%) studies reported on high grade irAE; 43 studies reported general high grade irAE and three studies reported on specific high grade irAE types. Endocrine irAE and pulmonary irAE were reported in 31 studies, gastrointestinal in 26, renal in 13, cardiac in 11, skin in 7, musculoskeletal in three, neurological in one study. Definition of irAE varied across included studies. Common Terminology Criteria for Adverse Events (versions 2, 3, 4 and 5) were the most widely used resource to define irAE among included studies.

Cardiac irAE included myocarditis, acute vascular events, pericardial disease, atrial fibrillation, cardiac failure, pericardial effusion, vasculitis, and dyspnoea. Endocrine irAE included hypo- and hyperthyroidism, adrenal insufficiency, hypophysitis, thyroiditis, isolated adrenocorticotropic hormone deficiency, new onset and worsening type 2 diabetes. Gastrointestinal irAE predominately included diarrhoea and colitis, and enteritis, pancreatitis, hepatitis. Musculoskeletal irAE included myositis alone or overlap manifestation (myositis and myocarditis and/or myasthenia gravis), inflammatory arthritis, arthralgia, and polymyalgia rheumatica. Neurological irAE included myasthenia gravis, neuropathy, Guillain-Barre syndrome, meningitis, encephalitis, myelitis, and demyelinating disorders. Pulmonary irAE predominately included pneumonitis and interstitial lung disease. Renal irAE included acute kidney injury, kidney failure and nephritis. Skin irAE included pruritus, rash, erythema, vitiligo, and skin eruption (macular-papular or eczematous). Full details of irAE types is provided in Supplementary Table S3.

### Event rates of immune-related adverse events

Event rates were calculated for 269 studies representing 37,442 patients with irAE among 278,772 total patients who received ICI. Event rate could not be calculated for 24 studies due to insufficient reporting on number of patients with irAE (14 studies), study population on ICI (8 studies), or neither number of patients with irAE nor study population on ICI (3 studies).

**Fig. 2:**
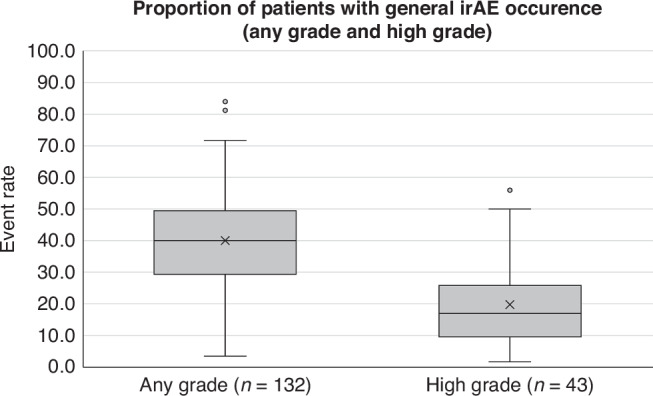
Box and whisker distribution of general irAE occurrence for any grade and high grade among included studies. *n* = *175 studies*. Any grade: minimum 3.5%; first quartile 29.3%; median 40.0%; third quartile 49.4%; maximum 84.0%; mean 40.0%; interquartile range 20.1%. High grade: minimum 1.7%; first quartile 9.6%; median 16.9%; third quartile 25.8%; maximum 56.0%; mean 19.7%; interquartile range 16.2%.

Event rate was calculated for 132 (*n* = 75,988) and 43 (*n* = 25,607) studies that reported either general and high grade irAE occurrence, respectively. The mean event rate for general irAE occurrence across any grade was 40.0% (37.3–42.7%; 95% CI) and high grade was 19.7% (15.8–23.7%; 95% CI). Figure 2 shows the distribution of general irAE occurrence for any grade and high grade among studies.

**Fig. 3:**
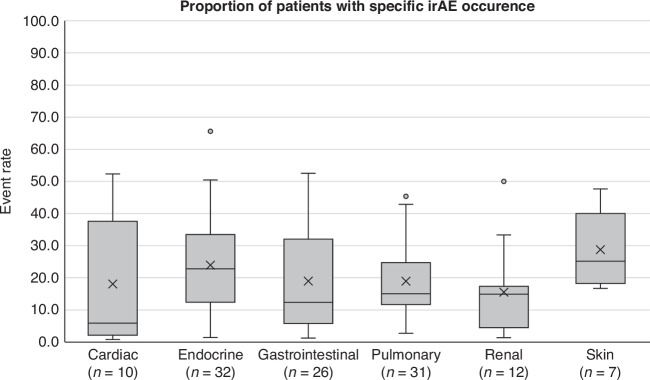
Box and whisker distribution of specific irAE occurrence for any grade and high grade among included studies. *n* = *118 studies*. Cardiac: minimum 0.8%; first quartile 2.2%; median 5.9%; third quartile 37.6%; maximum 52.3%; mean 18.0%; interquartile range 35.4%. Endocrine: minimum 1.5%; first quartile 12.4%; median 22.8%; third quartile 33.5%; maximum 65.6%; mean 23.9%; interquartile range 21.0%. Gastrointestinal: minimum 1.3%; first quartile 5.7%; median 12.5%; third quartile 33.3%; maximum 52.5%; mean 19.4%; interquartile range 27.6%. Pulmonary: minimum 2.8%; first quartile 11.7%; median 15.0%; third quartile 24.7%; maximum 45.4%; mean 18.9%; interquartile range 13.0%. Renal: minimum 1.4%; first quartile 4.5%; median 14.9%; third quartile 17.3%; maximum 50.0%; mean 15.5%; interquartile range 12.8%. Skin: minimum 16.7%; first quartile 18.2%; median 25.1%; third quartile 40%; maximum 47.7%; mean 28.7%; interquartile range 21.8%. Note: Due to low number of studies, event rates are not presented for musculoskeletal and neurologic irAE.

Overall, event rates were calculated for 121 studies (*n* = 188,506) that reported specific irAE occurrence. The mean event rates for each type of specific irAE occurrence were as follows: cardiac irAE 18.0% (5.9–30.2%; 95% CI), endocrine irAE 23.9% (18.8–29.1%; 95% CI), gastrointestinal irAE 19.4% (14.1–24.6%; 95% CI), pulmonary irAE 18.9% (15.1–22.7%; 95% CI), renal irAE 15.5% (7.3–23.7%; 95% CI), and skin irAE 28.7% (22.9–34.6%; 95% CI). Figure 3 shows the distribution of specific irAE occurrence among studies. Due to low number of studies, event rates are not presented for musculoskeletal and neurological irAE.

**Fig. 4:**
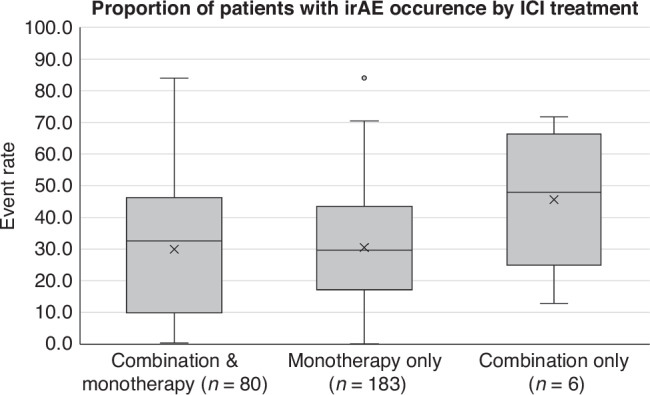
Box and whisker distribution of specific irAE occurrence for different ICI treatment types. *n = 269 studies*. Monotherapy: minimum 0.1%; first quartile 17.1%; median 29.7%; third quartile 43.5%; maximum 84.0%; mean 30.5%; interquartile range 26.4%. Combination: minimum 12.8%; first quartile 24.9%; median 48.0%; third quartile 66.3%; maximum 71.7%; mean 45.7%; interquartile range 41.4%. Combination & monotherapy: minimum 0.4%; first quartile 9.8%; median 32.6%; third quartile 46.3%; maximum 84.0%; mean 30.0%; interquartile range 36.5%.

Event rates varied across different ICI treatment types among 183 studies (*n* = 168,132) that focused on monotherapy, six (*n* = 339) combination therapy, and 80 studies (*n* = 110,301) that included both monotherapy and combination therapy. The mean event rate was expectedly lowest in monotherapy, 30.5% (28.1–32.9%; 95% CI), and highest with combination therapy, 45.7% (29.6–61.7%). Studies that had both monotherapy and combination therapy cohorts had a mean event rate of 30.0% (25.3–34.6%; 95% CI). Figure 4 shows the distribution of overall irAE occurrence across different ICI treatments among included studies.

### Association between irAE and proposed risk factors

Of the 293 studies analysed, 225 (76.8%) reported a difference, association, or correlation between the presence or absence of a specific factor, characteristic, or measure and the development of irAE at any stage prior to or during treatment. In contrast, 68 studies (23.2%) found no such relation. Further breakdown of the studies revealed that 158 (53.9%) specifically aimed to investigate the relation between a risk factor or predictor and the occurrence or severity. Meanwhile, 135 studies (46.1%) did not have this aim but contained data comparing the occurrence or severity of irAE between patients who did and did not experience irAE. It is worth noting that the statistical methods employed to assess differences, associations, or correlations varied considerably across these studies.

## Discussion

The immune-related adverse effects associated with checkpoint inhibitor immunotherapy is a growing research area and has implications for patients, clinicians, and healthcare services. In understanding these concerns, our systematic approach sought to identify event rates for irAE across various cancer indications, encompassing both ICI monotherapy and combination therapy in original research investigating risk factors or predictors. The findings of this study contribute to the understanding of the relative frequencies of various irAE and consolidates a large volume of studies and patient populations. It confirms that real-world data is comparable to irAE incidence reported in the clinical trial setting.

General irAE occurrence identified in this study reflects the prevalence or cumulative rates reported in cohort studies that include various cancer types and treatment types, such as Fujii (2018) and Yoshikawa (2022), where authors reported 34% and 27% of patients, respectively, who developed irAE across any grade [24, 25]. In a large meta-analysis, high grade irAE were reported in 27% of patients who received anti-CTLA and 17% on anti-PD-1 or -PD-L1 therapy [26]. This is comparable to the overall rate of high grade irAE found in the present study. The relative difference between event rates for high grade and general irAE emphasises the prominence of lower grade toxicity that patients are likely to experience. While fewer patients may experience severe toxicities that can pose barriers to treatment continuation and potentially cancer survival outcomes, lower grade irAE can cause persistent, long-term health conditions, particularly chronic endocrine and skin toxicities that require ongoing management. With small sample sizes, many studies have implicitly captured common irAE, such as gastrointestinal irAE, and were insufficiently sized to detect rare types of irAE. Risk factors for low prevalence irAE, such as neurological and ocular irAE, may remain under-reported.

Cardiac irAE may have a large distribution due to the diverse presentations included among studies, from dyspnoea to major cardiac adverse events. Endocrine, gastrointestinal, and skin irAE were more commonly located in this systematic review compared to pulmonary and renal irAE. The low number of studies identified for neurological irAE reflects the rare occurrence of these toxicities. It is worth noting that specific irAE such as myositis were recognised as musculoskeletal or neurological in different studies.

The findings of this study are compatible with comparative toxicity rates between combination therapy compared to monotherapy. Combination therapy is well-recognised to produce more frequent and higher grade irAE compared to ICI monotherapy [27]. For studies that included combination and monotherapy cohorts, reporting on irAE occurrence within individual treatment types was not consistently reported across all studies therefore it was not possible to present this in a systematic way.

As our understanding of irAE occurrence continues to develop, both with respect to real-world incidence and factors that may contribute to risk, there are several implications to clinical practice. Estimating irAE risk may help to identify which patients require greater surveillance/monitoring for treatment-related complications and holds an opportunity to stratify the use of scarce healthcare resources towards individualised care and tailored patient education. However, there are currently no validated risk factors that can be applied in the clinical setting to estimate the general propensity of an individual patient developing an irAE. There is also no clinical evidence to support treatment selection or modification based on irAE risk. Innovative methods, such as patient-reported outcome measures [28, 29] and methods to detect irAE in clinical records [30, 31], demonstrate the value of recognising irAE earlier during ICI treatment. These methods may also enhance reporting of lower severity irAE that can have persisting, long-term impacts on the quality of life for patients who receive ICI.

The primary limitation of our study stems from the systematic search criteria, which specifically targeted original research reporting on risk factors or predictors of irAE. These included studies detailing patient characteristics for groups both with and without irAE. While this approach resulted in a substantial number of studies being included in our analysis, it is possible that other relevant studies detailing patient characteristics but not meeting our specific search criteria were inadvertently excluded, particularly if studies were non-English or case reports/series. Included studies varied in study design and statistical analysis used to assess risk factors and predictors with irAE occurrence/severity, imparting high degree of heterogeneity. Consequently, it was not possible to conduct a systematic quality assessment across all studies. Variation in capturing population characteristics may introduce bias in irAE reporting and issues with external validity, as findings may not be generalisable to the whole cancer population. Definition of adverse events also varied across studies and different versions of CTCAE were used. Combined with subjectivity in detection, diagnosis and management of irAE, this may influence reporting at an institutional and clinician level, particularly in retrospective studies. Musculoskeletal adverse events appear to be under-represented in event rate calculation across studies despite being a common patient experience with ICI therapies. This may be due to lack of standardisation of irAE terminology and grading for some types of adverse events.

It is important to ensure that patient populations and irAE occurrence is comparable to existing literature as research on risk factors or predictors of irAE emerge. This study achieves this comparison and supports the growing body of evidence on the high prevalence of irAE in the real-world setting. Awareness of the frequency of irAE is useful for contextualising emerging research into irAE risk factors given the varying incidence of different irAE by organ system, cancer and treatment type.

## Conclusion

This systematic review summarises the characteristics among current peer-reviewed research that examines risk factors and predictors of irAE across cancer indications and ICI treatments and quantifies irAE occurrence across a large ICI patient population. This study supports and strengthens the understanding of the higher prevalence of irAE occurrence in the real-world setting highlighting that risk is an important consideration prior to commencement of ICI therapy. As immunotherapies are gaining traction for use in an expanding array of cancer types, it becomes paramount to gain a nuanced understanding of the risk factors and predictors of irAE. Our systematic review offers a comprehensive overview of current peer-reviewed research on risk factors and predictors of irAE across various cancer indications and ICI treatments. By quantifying the occurrence of irAE in a vast ICI patient population, this study substantiates the existing understanding and underscores the true prevalence of irAE in real-world settings. Further studies may be beneficial in refining guidelines and best practices for patient selection and monitoring during ICI treatment. Given these findings, clinicians should be aware and consider the associated risks when commencing ICI therapy and have a standard approach to discuss risks of adverse events with patients.

## Supplementary information

Table S1 Databases, search strategy and PICOS process

Table S2 Characteristics of all included studies and event rate

Table S3 Specific adverse events within each irAE type

## Data Availability

All data supporting the findings of this study are available within the paper and its Supplementary Material. Number of patients with irAE and total number of patients on ICI that were used to calculate event rate are provided in Supplementary Table S2, along with original reference.
