# Correction: Cancer and treatment specific incidence rates of immune-related adverse events induced by immune checkpoint inhibitors: a systematic review

**DOI:** 10.1038/s41416-024-02920-3

**Published:** 2024-12-10

**Authors:** Bishma Jayathilaka, Farah Mian, Fanny Franchini, George Au-Yeung, Maarten IJzerman

**Affiliations:** 1 Pharmacy Department, Peter MacCallum Cancer Centre, Melbourne, VIC Australia; 2 Sir Peter MacCallum Department of Oncology, Faculty of Medicine, Dentistry and Health Sciences, University of Melbourne, Melbourne, VIC Australia; 3 Cancer Health Services Research Unit, Centre for Cancer Research, Faculty of Medicine, Dentistry and Health Sciences, University of Melbourne, Melbourne, VIC Australia; 4 Department of Medical Oncology, Peter MacCallum Cancer Centre, Melbourne, VIC Australia; 5 Erasmus School of Health Policy & Management, Erasmus University, Rotterdam, The Netherlands

**Keywords:** Cancer immunotherapy, Adverse effects, Epidemiology

Correction to: *British Journal of Cancer* 10.1038/s41416-024-02887-1, published online 03 November 2024

In this article values in the sentence “Mean event rate for ICI monotherapy was 29.7% (27.7–33.2%), 45.7% (29.6–61.7%) for ICI combination therapy, and 30.3% (27.3–35.0%) for both ICI monotherapy and combination therapy.” are given erroneously. They should read “Mean event rate for ICI monotherapy was 30.5% (28.1–32.9%), 45.7% (29.6–61.7%) for ICI combination therapy, and 30.0% (25.3–34.6%) for both ICI monotherapy and combination therapy.”

The original article has been corrected.

