# Supplementary material for: Cancer and treatment specific incidence rates of immune-related adverse events induced by immune checkpoint inhibitors: a systematic review
Source: Br J Cancer. 2024 Nov 3;132(1):51–7. doi: 10.1038/s41416-024-02887-1 (PMC11723908; doi:10.1038/s41416-024-02887-1)
Supplement: Supplementary file 1 — Table S1 Databases, search strategy and PICOS process [file 41416_2024_2887_MOESM1_ESM.docx]

**Supplementary Table S1:** Databases, search strategy and PICOS process.

Search date: 23^rd^ September 2021 (additional papers 18^th^ January 2022).

| **Ovid MEDLINE(R) ALL 1946 to Present; Embase Classic+Embase 1947 to Present** | |
| --- | --- |
| 1 | (exp Immune Checkpoint Inhibitors/) OR checkpoint inhibit* OR ipilimumab OR tremelimumab OR pembrolizumab OR nivolumab OR cemiplimab OR tislelizumab OR dostarlimab OR spartalizumab OR sintilimab OR atezolizumab OR avelumab OR PD1 OR PD-1 OR PDL1 OR PD-L1 OR CTLA4 OR CTLA-4.mp |
| 2 | (immune-related adverse event* OR irAE*) OR toxic* OR adverse event*.mp |
| 3 | risk factor* OR predict*.mp |
| 4 | 1 AND 2 AND 3 |
| 5 | exp COVID-19/ OR exp Vaccines/ |
| 6 | 4 NOT 5 |
| 7 | limit (English language and yr=”2017-2021”) |
| **Web of Science Core Collection <All Editions>** | |
| (((TS=("Checkpoint Inhibit*" OR Ipilimumab OR Tremelimumab OR Pembrolizumab OR Nivolumab OR Cemiplimab OR Tislelizumab OR Dostarlimab OR Spartalizumab OR Sintilimab OR Atezolizumab OR Avelumab OR Durvalumab)) AND TS=(toxic* OR "adverse event*" OR "immune related adverse event* OR irAE*")) AND TS=("risk factor*" OR predict*)) NOT TS=(vaccin* OR "covid-19") and 2021 or 2020 or 2019 or 2018 or 2017 (Publication Years) | |
| **PICOS process** | |
| P | Adult patients with cancer with immune-related adverse event/s |
| I | Receiving immune checkpoint inhibitor treatment |
| C | No immune-related adverse event/s |
| O | Differences in population characteristics across treatments and cancers |
| S | Original research (including case series, case-control studies, observational studies, cohort studies, pharmacovigilance studies, randomised controlled trials, systematic reviews, and meta-analyses) |
