# Supplementary material for: Cancer and treatment specific incidence rates of immune-related adverse events induced by immune checkpoint inhibitors: a systematic review
Source: Br J Cancer. 2024 Nov 3;132(1):51–7. doi: 10.1038/s41416-024-02887-1 (PMC11723908; doi:10.1038/s41416-024-02887-1)
Supplement: Supplementary file 2 — Table S2 Characteristics of all included studies and event rate [file 41416_2024_2887_MOESM2_ESM.docx]

**Supplementary Table S2:** Characteristics of all included studies and event rate (if calculated). *n = 293*

| **Author Year** | **Study Title** | **Study Design** | **Cancer Type** | **Cancer Stage (if specified)** | **ICI Type(s)** | **Specific ICI Agents** | **Patients With irAE** | **ICI Patient Population** | **Event Rate (%)** | **Type of irAE** |
| --- | --- | --- | --- | --- | --- | --- | --- | --- | --- | --- |
| Abdel-Wahab 2018 | Use of immune checkpoint inhibitors in the treatment of patients with cancer and preexisting autoimmune disease: A systematic review. | Systematic review | Pan-cancer | Advanced or metastatic | Combination & monotherapy | Anti-PD-1; Anti-PD-L1; Nivolumab; Pembrolizumab; Atezolizumab; Ipilimumab; Combination Ipilimumab + Nivolumab | 42 | 123 | 34.1 | General irAE |
| Abdel-Wahab 2021 | Genetic determinants of immune-related adverse events in patients with melanoma receiving immune checkpoint inhibitors. | Retrospective Single-centre  Case-control study | Melanoma | Adjuvant, III, IV | Combination & monotherapy | Nivolumab; Pembrolizumab; Atezolizumab; Ipilimumab; Combination Ipilimumab + Nivolumab; Combination Ipilimumab + Pembrolizumab | 44 | 89 | 49.4 | General irAE |
| Abdelrahim 2021 | Incidence, predictors, and survival impact of acute kidney injury in patients with melanoma treated with immune checkpoint inhibitors: A 10-year single-institution analysis. | Retrospective Single-centre Cohort study | Melanoma | Adjuvant, advanced or metastatic | Combination & monotherapy | Nivolumab; Pembrolizumab; Atezolizumab; Ipilimumab; Combination Ipilimumab + Nivolumab; Combination Ipilimumab + Pembrolizumab; Dostarlimab | 130 | Not specified | Not assessable | Renal |
| Abu-Sbeih 2019 (1) | Clinical characteristics and outcomes of immune checkpoint inhibitor-induced pancreatic injury. | Retrospective Single-centre Cohort study | Pan-cancer | III, IV | Combination & monotherapy | Anti-PD-1; Anti-PD-L1; Anti-CTLA-4; Combination Anti-PD1/L1 + Anti-CTLA-4 | 82 | 2279 | 3.6 | Gastrointestinal (grade specific) |
| Abu-Sbeih 2019 (2) | Immune checkpoint inhibitor-induced colitis as a predictor of survival in metastatic melanoma. | Retrospective Single-centre  Case-control study | Melanoma | III, IV M1a-c | Combination & monotherapy | Anti-PD-1; Anti-PD-L1; Anti-CTLA-4; Combination Anti-PD1/L1 + Anti-CTLA-4 | 173 | 1983 | 8.7 | Gastrointestinal |
| Abu-Sbeih 2019 (3) | Impact of antibiotic therapy on the development and response to treatment of immune checkpoint inhibitor-mediated diarrhea and colitis. | Retrospective Single-centre Cohort study | Pan-cancer | III, IV | Combination & monotherapy | Anti-PD-1; Anti-PD-L1; Anti-CTLA-4; Combination Anti-PD1/L1 + Anti-CTLA-4 | 434 | 826 | 52.5 | Gastrointestinal |
| Abu-Sbeih 2020 | Immune checkpoint inhibitor therapy in patients with preexisting inflammatory bowel disease. | Retrospective  Multi-centre  Cohort study | Pan-cancer | III, IV | Combination & monotherapy | Anti-PD-1; Anti-PD-L1; Anti-CTLA-4; Combination Anti-PD1/L1 + Anti-CTLA-4 | 42 | 102 | 41.2 | Gastrointestinal |
| **Author Year** | **Study Title** | **Study Design** | **Cancer Type** | **Cancer Stage (if specified)** | **ICI Type(s)** | **Specific ICI Agents** | **Patients With irAE** | **ICI Patient Population** | **Event Rate (%)** | **Type of irAE** |
| Akamatsu 2020 | Immune-related adverse events by immune checkpoint inhibitors significantly predict durable efficacy even in responders with advanced non-small cell lung cancer. | Prospective  Single-centre  Cohort study | Non-small cell lung cancer | Advanced | Monotherapy | Nivolumab; Pembrolizumab; Atezolizumab | 15 | Not specified | Not assessable | General irAE |
| Al Ashi 2021 | Endocrine toxicity and outcomes in patients with metastatic malignancies treated with immune checkpoint inhibitors. | Retrospective Single-centre Observational study | Pan-cancer | Metastatic | Monotherapy | Nivolumab; Pembrolizumab; Atezolizumab; Avelumab; Durvalumab; Ipilimumab | 98 | 551 | 17.8 | Endocrine |
| Aldrich 2021 | Inflammatory myositis in cancer patients receiving immune checkpoint inhibitors. | Retrospective Single-centre Cohort study | Pan-cancer | Not specified | Combination & monotherapy | Combination Anti-PD1/L1 + Anti-CTLA-4; Nivolumab; Pembrolizumab; Cemiplimab; Atezolizumab; Avelumab; Durvalumab; Ipilimumab | 36 | 9088 | 0.4 | Musculoskeletal |
| Almutairi 2021 | Association of immune-checkpoint inhibitors and the risk of immune-related colitis among elderly patients with advanced melanoma: Real-world evidence from the SEER-Medicare database. | Retrospective Cohort study | Melanoma | III, IV | Monotherapy | Nivolumab; Pembrolizumab; Ipilimumab | Not specified | 274 | Not assessable | Gastrointestinal |
| Alves 2021 | Peripheral blood eosinophilia may be a prognostic biomarker in non-small cell lung cancer patients treated with immunotherapy. | Retrospective Single-centre Cohort study | Non-small cell lung cancer | IIIb, IV | Monotherapy | Nivolumab; Pembrolizumab; Atezolizumab | Not specified | 121 | Not assessable | General irAE |
| Andrews 2021 | Gut microbiota signatures are associated with toxicity to combined CTLA-4 and PD-1 blockade. | Prospective  Single-centre Experimental study | Melanoma | III, IV | Combination only | Combination Anti-PD-1 + Anti-CTLA-4 | 38 | 77 | 49.4 | High grade irAE |
| Arakawa 2019 | Clonality of CD4+ blood T cells predicts longer survival with CTLA4 or PD-1 checkpoint inhibition in advanced melanoma. | Prospective  Single-centre  Cohort study | Melanoma | M1a-c | Monotherapy | Pembrolizumab; Ipilimumab | 4 | 20 | 20.0 | Gastrointestinal (including high grade) |
| Archibald 2020 | Immune checkpoint inhibitors in older adults with melanoma or cutaneous malignancies: The Wilmot Cancer Institute experience. | Retrospective Single-centre Cohort study | Melanoma | Not specified | Monotherapy | Nivolumab; Pembrolizumab; Ipilimumab | 46 | 120 | 38.3 | General irAE |
|  |  |  |  |  |  |  | 26 |  | 21.7 | High grade irAE |
| Asada 2021 | The risk factors associated with immune checkpoint inhibitor-related pneumonitis. | Retrospective Pharmacovigilance study | Pan-cancer | Not specified | Monotherapy | Nivolumab; Pembrolizumab; Cemiplimab; Atezolizumab; Avelumab; Durvalumab; Tremelimumab | 1414 | 51166 | 2.8 | Pulmonary |

| **Author Year** | **Study Title** | **Study Design** | **Cancer Type** | **Cancer Stage (if specified)** | **ICI Type(s)** | **Specific ICI Agents** | **Patients With irAE** | **ICI Patient Population** | **Event Rate (%)** | **Type of irAE** |
| --- | --- | --- | --- | --- | --- | --- | --- | --- | --- | --- |
| Aso 2020 | Association between skin reaction and clinical benefit in patients treated with anti-programmed cell death 1 monotherapy for advanced non-small cell lung cancer. | Retrospective Single-centre Cohort study | Non-small cell lung cancer | Advanced | Monotherapy | Nivolumab; Pembrolizumab | 51 | 155 | 32.9 | Skin |
| Awadalla 2019 | Influenza vaccination and myocarditis among patients receiving immune checkpoint inhibitors. | Retrospective Single-centre  Case-control study | Pan-cancer | Not specified | Combination & monotherapy | Nivolumab; Pembrolizumab; Atezolizumab; Avelumab; Durvalumab; Ipilimumab; Combination Ipilimumab + Nivolumab; Combination Ipilimumab + Pembrolizumab; Tremelimumab; Combination Tremelimumab + Durvalumab; Combination Tremelimumab + Avelumab | 101 | 302 | 33.4 | Cardiac |
| Awadalla 2020 | Global longitudinal strain and cardiac events in patients with immune checkpoint inhibitor-related myocarditis. | Retrospective Single-centre  Case-control study | Pan-cancer | Not specified | Combination & monotherapy | Anti-PD-1; Anti-PD-L1; Anti-CTLA-4; Combination Anti-PD1/L1 + Anti-CTLA-4 | 101 | 193 | 52.3 | Cardiac |
| Bai 2021 (1) | Analysis of characteristics and predictive factors of immune checkpoint inhibitor-related adverse events. | Retrospective Single-centre Cohort study | Pan-cancer | III, IV | Combination & monotherapy | Nivolumab; Pembrolizumab; Atezolizumab; Combination Ipilimumab + Nivolumab; Sintilimab; Tislelizumab ; Toripalimab, Camrelizumab | 41 | 105 | 39.0 | General irAE |

| **Author Year** | **Study Title** | **Study Design** | **Cancer Type** | **Cancer Stage (if specified)** | **ICI Type(s)** | **Specific ICI Agents** | **Patients With irAE** | **ICI Patient Population** | **Event Rate (%)** | **Type of irAE** |
| --- | --- | --- | --- | --- | --- | --- | --- | --- | --- | --- |
| Bai 2021 (2) | Common immune-related adverse events of immune checkpoint inhibitors in the gastrointestinal system: A study based on the US Food and Drug Administration Adverse Event Reporting System. | Retrospective Pharmacovigilance study | Pan-cancer | Not specified | Combination & monotherapy | Anti-PD-1; Anti-PD-L1; Anti-CTLA-4; Combination Anti-PD1/L1 + Anti-CTLA-4; Nivolumab; Pembrolizumab; Cemiplimab; Atezolizumab; Avelumab; Durvalumab; Ipilimumab; Combination Ipilimumab + Nivolumab; Combination Ipilimumab + Pembrolizumab | Not specified | Not specified | Not assessable | Gastrointestinal |
| Baldini 2020 | Impact of aging on immune-related adverse events generated by anti-programmed death (ligand)PD-(L)1 therapies. | Retrospective Single-centre Cohort study | Pan-cancer | Not specified | Monotherapy | Nivolumab; Pembrolizumab; Atezolizumab; Avelumab | 165 | 603 | 27.4 | General irAE |
|  |  |  |  |  |  |  | 58 |  | 9.6 | High grade irAE |
| Bar 2019 | Acute vascular events as a possibly related adverse event of immunotherapy: A single-institute retrospective study. | Retrospective Single-centre Cohort study | Pan-cancer | Not specified | Combination & monotherapy | Combination Anti-PD1/L1 + Anti-CTLA-4; Nivolumab; Pembrolizumab; Atezolizumab; Durvalumab; Ipilimumab | 37 | 1215 | 3.0 | Cardiac |
| Barrón 2020 | Risk of developing checkpoint immune pneumonitis and its effect on overall survival in non-small cell lung cancer patients previously treated with radiotherapy. | Retrospective Single-centre Cohort study | Non-small cell lung cancer | III, IV | Monotherapy | Nivolumab; Pembrolizumab | 22 | 101 | 21.8 | Pulmonary (grade specific) |
| Basak 2020 | Overt thyroid dysfunction and anti-thyroid antibodies predict response to anti-PD-1 immunotherapy in cancer patients. | Prospective  Single-centre  Cohort study | Melanoma; Non-small cell lung cancer; Renal cell carcinoma | Advanced or metastatic | Monotherapy | Nivolumab; Pembrolizumab | 54 | 168 | 32.1 | Endocrine |
| Bastacky 2021 | Immune-related adverse events in PD-1 treated melanoma and impact upon anti-tumor efficacy: A real world analysis. | Retrospective Single-centre Cohort study | Melanoma | Unresectable stage III, M1a-d | Monotherapy | Nivolumab; Pembrolizumab | 114 | 190 | 60.0 | General irAE |
| Betof 2017 | Impact of age on outcomes with immunotherapy for patients with melanoma. | Retrospective  Multi-centre  Cohort study | Melanoma | Metastatic | Monotherapy | Anti-PD-1; Anti-PD-L1 | 110 | 254 | 43.3 | General irAE |
| Biewenga 2021 | Checkpoint inhibitor induced hepatitis and the relation with liver metastasis and outcome in advanced melanoma patients. | Retrospective  Multi-centre  Cohort study | Melanoma | Advanced | Combination & monotherapy | Nivolumab; Pembrolizumab; Ipilimumab; Combination Ipilimumab + Nivolumab | 139 | 2561 | 5.4 | Gastrointestinal (grade specific) |

| **Author Year** | **Study Title** | **Study Design** | **Cancer Type** | **Cancer Stage (if specified)** | **ICI Type(s)** | **Specific ICI Agents** | **Patients With irAE** | **ICI Patient Population** | **Event Rate (%)** | **Type of irAE** |
| --- | --- | --- | --- | --- | --- | --- | --- | --- | --- | --- |
| Bins 2018 | Association between single-nucleotide polymorphisms and adverse events in nivolumab-treated non-small cell lung cancer patients. | Retrospective  Multi-centre  Cohort study | Non-small cell lung cancer | Not specified | Monotherapy | Nivolumab | 181 | 322 | 56.2 | General irAE |
| Booka 2021 | Impact of immune-related adverse events on nivolumab efficacy in patients with upper gastrointestinal cancer. | Retrospective Single-centre Cohort study | Upper gastrointestinal cancer | Not specified | Monotherapy | Nivolumab | 13 | 50 | 26.0 | General irAE |
| Bottlaender 2020 | Cutaneous adverse events: A predictor of tumour response under anti-PD-1 therapy for metastatic melanoma, a cohort analysis of 189 patients. | Retrospective Single-centre Observational cohort study | Melanoma | IV | Monotherapy | Nivolumab; Pembrolizumab | 39 | 189 | 20.6 | Skin |
| Brilli 2021 | Baseline serum TSH levels predict the absence of thyroid dysfunction in cancer patients treated with immunotherapy. | Retrospective Single-centre Cohort study | Pan-cancer | Metastatic or unresectable advanced | Combination & monotherapy | Anti-PD-1; Anti-PD-L1; Combination Anti-PD-1 + Anti-CTLA-4 | 11 | 68 | 16.2 | Endocrine |
| Brown 2021 | Combination anti-PD1 and ipilimumab therapy in patients with advanced melanoma and pre-existing autoimmune disorders. | Retrospective  Multi-centre  Cohort study | Melanoma | M1a-d | Combination only | Combination Ipilimumab + Nivolumab; Combination Ipilimumab + Pembrolizumab | 37 | 55 | 67.3 | General irAE |
| Byrne 2021 | Immune-related adverse events in cancer patients being treated with immune checkpoint inhibitors. | Retrospective Single-centre Cohort study | Pan-cancer | Not specified | Combination & monotherapy | Nivolumab; Pembrolizumab; Atezolizumab; Combination Ipilimumab + Nivolumab | 57 | 131 | 43.5 | General irAE |
| Chan 2020 | Higher checkpoint inhibitor arthritis disease activity may be associated with cancer progression: Results from an observational registry. | Prospective  Single-centre Observational study | Pan-cancer | III, IV | Combination & monotherapy | Anti-PD-1; Anti-PD-L1; Combination Anti-PD1/L1 + Anti-CTLA-4 | 42 | 66 | 63.6 | Musculoskeletal |
| Chaput 2017 | Baseline gut microbiota predicts clinical response and colitis in metastatic melanoma patients treated with ipilimumab. | Prospective  Single-centre  Cohort study | Melanoma | M1a-c | Monotherapy | Ipilimumab | 7 | 38 | 18.4 | Gastrointestinal |
| Chen 2020 | Renal adverse effects following the use of different immune checkpoint inhibitor regimens: A real-world pharmacoepidemiology study of post-marketing surveillance data. | Retrospective Pharmacovigilance study | Pan-cancer | Not specified | Combination & monotherapy | Nivolumab; Pembrolizumab; Atezolizumab; Avelumab; Durvalumab; Ipilimumab; Combination Ipilimumab + Nivolumab; Combination Ipilimumab + Pembrolizumab | 1444 | Not specified | Not assessable | Renal |

| **Author Year** | **Study Title** | **Study Design** | **Cancer Type** | **Cancer Stage (if specified)** | **ICI Type(s)** | **Specific ICI Agents** | **Patients With irAE** | **ICI Patient Population** | **Event Rate (%)** | **Type of irAE** |
| --- | --- | --- | --- | --- | --- | --- | --- | --- | --- | --- |
| Chen 2021 | Cardiotoxicity induced by immune checkpoint inhibitors: A pharmacovigilance study from 2014 to 2019 based on FAERS. | Retrospective Pharmacovigilance study | Pan-cancer | Not specified | Monotherapy | Nivolumab; Pembrolizumab; Cemiplimab; Atezolizumab; Avelumab; Durvalumab; Ipilimumab | 9271 | Not specified | Not assessable | Cardiac |
| Cheung 2020 | Immune checkpoint inhibitor-related colitis assessment and prognosis: Can IBD scoring point the way? | Retrospective  Multi-centre Observational cohort study | Melanoma; Non-small cell lung cancer; Renal/urothelial cancer; (Pan-cancer approach) | Metastatic | Combination & monotherapy | Nivolumab; Pembrolizumab; Ipilimumab; Combination Ipilimumab + Nivolumab | 134 | 1074 | 12.5 | Gastrointestinal (grade specific) |
| Chmielewska 2021 | Do endocrine adverse events predict longer progression-free survival among patients with non-small-cell lung cancer receiving nivolumab?. | Retrospective Single-centre Cohort study | Non-small cell lung cancer | IIIb, IV | Monotherapy | Nivolumab | 12 | 35 | 34.3 | Endocrine |
| Cho 2018 | Characteristics, incidence, and risk factors of immune checkpoint inhibitor-related pneumonitis in patients with non-small cell lung cancer. | Retrospective Single-centre Cohort study | Non-small cell lung cancer | Not specified | Combination & monotherapy | Nivolumab; Pembrolizumab; Atezolizumab; Durvalumab; Ipilimumab; Combination Ipilimumab + Nivolumab | 22 | 167 | 13.2 | Pulmonary |
| Chu 2020 | Body composition is prognostic and predictive of ipilimumab activity in metastatic melanoma. | Retrospective Single-centre Cohort study | Melanoma | III, IV | Monotherapy | Ipilimumab | Not specified | 97 | Not assessable | General irAE |
| Collet 2021 | Association between body mass index and survival outcome in metastatic cancer patients treated by immunotherapy: Analysis of a French retrospective cohort. | Retrospective Single-centre Cohort study | Pan-cancer | Metastatic | Combination & monotherapy | Nivolumab; Pembrolizumab; Atezolizumab; Avelumab; Durvalumab; Ipilimumab; Combination Ipilimumab + Nivolumab; Combination Tremelimumab + Durvalumab | 112 | 272 | 41.2 | General irAE |
|  |  |  |  |  |  |  | 21 |  | 7.7 | High grade irAE |
| Corbaux 2019 | Older and younger patients treated with immune checkpoint inhibitors have similar outcomes in real-life setting. | Retrospective  Multi-centre  Cohort study | Pan-cancer | Advanced | Monotherapy | Anti-PD-L1; Anti-CTLA-4 | 207 | 410 | 50.5 | General irAE |
|  |  |  |  |  |  |  | 49 |  | 12.0 | High grade irAE |
| Correale 2020 | HLA expression correlates to the risk of immune checkpoint inhibitor-induced pneumonitis. | Retrospective  Multi-centre  Cohort study | Pan-cancer | Not specified | Monotherapy | Nivolumab; Pembrolizumab; Atezolizumab | 68 | 256 | 26.6 | General irAE |
|  |  |  |  |  |  |  | 29 |  | 11.3 | Pulmonary |

| **Author Year** | **Study Title** | **Study Design** | **Cancer Type** | **Cancer Stage (if specified)** | **ICI Type(s)** | **Specific ICI Agents** | **Patients With irAE** | **ICI Patient Population** | **Event Rate (%)** | **Type of irAE** |
| --- | --- | --- | --- | --- | --- | --- | --- | --- | --- | --- |
| Cortazar 2020 | Clinical features and outcomes of immune checkpoint inhibitor-associated AKI: A multicenter study. | Retrospective  Multi-centre  Case-control study | Pan-cancer | Not specified | Combination & monotherapy | Anti-PD-1; Anti-PD-L1; Anti-CTLA-4; Combination Anti-PD1/L1 + Anti-CTLA-4 | 138 | 414 | 33.3 | Renal |
| Cortellini 2019 (1) | Clinical outcomes of patients with advanced cancer and pre-existing autoimmune diseases treated with anti-programmed death-1 immunotherapy: A real-world transverse study. | Retrospective  Multi-centre Observational study | Pan-cancer | IV | Monotherapy | Nivolumab; Pembrolizumab | 322 | 751 | 42.9 | General irAE |
|  |  |  |  |  |  |  | 67 |  | 8.9 | High grade irAE |
| Cortellini 2019 (2) | Correlations between the immune-related adverse events spectrum and efficacy of anti-PD1 immunotherapy in NSCLC patients. | Retrospective  Multi-centre Observational study | Non-small cell lung cancer | Advanced | Monotherapy | Nivolumab; Pembrolizumab | 231 | 559 | 41.3 | General irAE |
| Cortellini 2019 (3) | A multicenter study of body mass index in cancer patients treated with anti-PD-1/PD-L1 immune checkpoint inhibitors: When overweight becomes favorable. | Retrospective  Multi-centre Observational study | Pan-cancer | IV | Monotherapy | Nivolumab; Pembrolizumab; Atezolizumab | 393 | 976 | 40.3 | General irAE |
|  |  |  |  |  |  |  | 63 |  | 6.5 | High grade irAE |
| Cortellini 2019 (4) | Predictive value of skeletal muscle mass for immunotherapy with nivolumab in non-small cell lung cancer patients: A "hypothesis-generator" preliminary report. | Retrospective Single-centre Observational study | Non-small cell lung cancer | IV | Monotherapy | Nivolumab | 14 | 23 | 60.9 | General irAE |
| Cortellini 2020 (1) | Another side of the association between body mass index (BMI) and clinical outcomes of cancer patients receiving programmed cell death protein-1 (PD-1)/ Programmed cell death-ligand 1 (PD-L1) checkpoint inhibitors: A multicentre analysis of immune-related. | Retrospective  Multi-centre Observational study | Pan-cancer | IV | Monotherapy | Nivolumab; Pembrolizumab; Atezolizumab | 419 | 1070 | 39.2 | General irAE |
|  |  |  |  |  |  |  | 67 |  | 6.3 | High grade irAE |
| Cortellini 2020 (2) | Evaluating the role of FAMIly history of cancer and diagnosis of multiple neoplasms in cancer patients receiving PD-1/PD-L1 checkpoint inhibitors: The multicenter FAMI-L1 study. | Retrospective  Multi-centre Observational study | Pan-cancer | IV | Monotherapy | Nivolumab; Pembrolizumab; Atezolizumab | 329 | 822 | 40.0 | General irAE |
| Cortellini 2020 (3) | Weighing the role of skeletal muscle mass and muscle density in cancer patients receiving PD-1/PD-L1 checkpoint inhibitors: A multicenter real-life study. | Retrospective  Multi-centre Observational study | Pan-cancer | Not specified | Monotherapy | Anti-PD-1; Anti-PD-L1; Nivolumab; Pembrolizumab; Atezolizumab | 25 | 100 | 25.0 | General irAE |
| Costantini 2018 | Predictive role of plasmatic biomarkers in advanced non-small cell lung cancer (NSCLC) treated with nivolumab (NIVO). | Prospective  Single-centre Exploratory study | Non-small cell lung cancer | I to IV | Monotherapy | Nivolumab | 8 | 43 | 18.6 | High grade irAE |

| **Author Year** | **Study Title** | **Study Design** | **Cancer Type** | **Cancer Stage (if specified)** | **ICI Type(s)** | **Specific ICI Agents** | **Patients With irAE** | **ICI Patient Population** | **Event Rate (%)** | **Type of irAE** |
| --- | --- | --- | --- | --- | --- | --- | --- | --- | --- | --- |
| Costantini 2021 | Plasma biomarkers screening by multiplex ELISA assay in patients with advanced non-small cell lung cancer treated with immune checkpoint inhibitors. | Prospective  Single-centre Exploratory study | Non-small cell lung cancer | I to IV | Monotherapy | Nivolumab; Pembrolizumab | 6 | 35 | 17.1 | High grade irAE |
| Cousin 2021 | Incidence, risk factors, and CT characteristics of radiation recall pneumonitis induced by immune checkpoint inhibitor in lung cancer. | Retrospective Single-centre Cohort study | Lung cancer | Advanced | Monotherapy | Nivolumab; Pembrolizumab; Atezolizumab | 15 | 80 | 18.8 | Pulmonary |
| Cunningham-Bussel 2021 | Predictors of rheumatic immune-related adverse events and de novo inflammatory arthritis after immune checkpoint inhibitor treatment for cancer. | Retrospective  Multi-centre  Case-control study | Pan-cancer | Not specified | Combination & monotherapy | Nivolumab; Pembrolizumab; Cemiplimab; Atezolizumab; Avelumab; Durvalumab; Ipilimumab; Combination Ipilimumab + Nivolumab; Combination Ipilimumab + Pembrolizumab; Tremelimumab; Combination Tremelimumab + Durvalumab | 226 | 8028 | 2.8 | Musculoskeletal |
| Cybulska-Stopa 2019 | Immune checkpoint inhibitors therapy in older patients (>= 70 years) with metastatic melanoma: A multicentre study. | Retrospective Single-centre Cohort study | Melanoma | Metastatic | Monotherapy | Nivolumab; Pembrolizumab; Ipilimumab | 82 | 290 | 28.3 | High grade irAE |
| Daly 2017 | The impact of body composition parameters on ipilimumab toxicity and survival in patients with metastatic melanoma. | Retrospective  Multi-centre  Cohort study | Melanoma | M1a-c | Monotherapy | Ipilimumab | 47 | 84 | 56.0 | High grade irAE |
| Danlos 2018 | Safety and efficacy of anti-programmed death 1 antibodies in patients with cancer and pre-existing autoimmune or inflammatory disease. | Retrospective Single-centre  Case-control study | Pan-cancer | III, IV | Monotherapy | Nivolumab; Pembrolizumab; Avelumab | 20 | 45 | 44.4 | General irAE |
| Das 2018 | Early B cell changes predict autoimmunity following combination immune checkpoint blockade. | Prospective  Single-centre  Cohort study | Melanoma | Advanced | Combination & monotherapy | Anti-PD-1; Anti-CTLA-4; Combination Anti-PD-1 + Anti-CTLA-4 | 12 | 39 | 30.8 | High grade irAE |
| De Filippi 2021 | Body mass index is not associated with survival outcomes and immune-related adverse events in patients with Hodgkin lymphoma treated with the immune checkpoint inhibitor nivolumab. | Retrospective  Multi-centre  Cohort study | Hodgkin's lymphoma | Mostly III-IV | Monotherapy | Nivolumab | 67 | 133 | 50.4 | General irAE |
|  |  |  |  |  |  |  | 26 |  | 19.5 | High grade irAE |

| **Author Year** | **Study Title** | **Study Design** | **Cancer Type** | **Cancer Stage (if specified)** | **ICI Type(s)** | **Specific ICI Agents** | **Patients With irAE** | **ICI Patient Population** | **Event Rate (%)** | **Type of irAE** |
| --- | --- | --- | --- | --- | --- | --- | --- | --- | --- | --- |
| de Moel 2019 | Autoantibody development under treatment with immune-checkpoint inhibitors. | Prospective  Single-centre  Cohort study | Melanoma | IIIc, IV | Monotherapy | Ipilimumab | 61 | 99 | 61.6 | General irAE |
| Delaunay 2017 | Immune-checkpoint inhibitors associated with interstitial lung disease in cancer patients. | Retrospective  Multi-centre  Cohort study | Pan-cancer | Mostly IV | Monotherapy | Anti-PD-1; Anti-PD-L1; Anti-CTLA-4 | 64 | 1826 | 3.5 | Pulmonary (including high grade) |
| Diehl 2017 | Relationships between lymphocyte counts and treatment-related toxicities and clinical responses in patients with solid tumors treated with PD-1 checkpoint inhibitors. | Retrospective Single-centre Cohort study | Pan-cancer | Not specified | Combination & monotherapy | Nivolumab; Pembrolizumab; Combination Ipilimumab + Nivolumab; Combination Ipilimumab + Pembrolizumab | 51 | 167 | 30.5 | General irAE |
|  |  |  |  |  |  |  | 15 |  | 9.0 | High grade irAE |
| Dothard 2020 | Performance status and age as predictors of immunotherapy outcomes in advanced non-small-cell lung cancer. | Retrospective Single-centre Cohort study | Non-small cell lung cancer | IV | Monotherapy | Nivolumab; Pembrolizumab; Atezolizumab; Ipilimumab | 29 | 285 | 10.2 | General irAE |
| Drobni 2020 | Decreased absolute lymphocyte count and increased neutrophil/lymphocyte ratio with immune checkpoint inhibitor-associated myocarditis. | Retrospective  Multi-centre  Case-control study | Pan-cancer | Not specified | Combination & monotherapy | Anti-PD-1; Anti-PD-L1; Anti-CTLA-4; Combination Anti-PD1/L1 + Anti-CTLA-4 | 55 | 110 | 50.0 | Cardiac |
| Duma 2019 | Sex differences in tolerability to anti-programmed cell death protein 1 therapy in patients with metastatic melanoma and non-small cell lung cancer: Are we all equal?. | Retrospective  Multi-centre  Cohort study | Melanoma; Non-small cell lung cancer | Advanced or metastatic | Monotherapy | Nivolumab; Pembrolizumab | 219 | 476 | 46.0 | General irAE |
| Dumenil 2018 | Clinical factors associated with early progression and grade 3-4 toxicity in patients with advanced non-small-cell lung cancers treated with nivolumab. | Retrospective  Multi-centre  Cohort study | Non-small cell lung cancer | IIIB, IV | Monotherapy | Nivolumab | 47 | 67 | 70.1 | General irAE |
|  |  |  |  |  |  |  | 28 |  | 41.8 | High grade irAE |
| Economopoulou 2020 | Association of autoimmunity with survival in patients with recurrent/metastatic head and neck squamous cell carcinoma treated with nivolumab. | Retrospective Single-centre Cohort study | Head and neck cancer | Recurrent or metastatic | Monotherapy | Nivolumab | 29 | 89 | 32.6 | General irAE |
| Egami 2021 (1) | Absolute lymphocyte count predicts immune-related adverse events in patients with non-small-cell lung cancer treated with nivolumab monotherapy: A multicenter retrospective study. | Retrospective  Multi-centre Observational study | Non-small cell lung cancer | Advanced | Monotherapy | Nivolumab | 73 | 171 | 42.7 | General irAE |
| Egami 2021 (2) | Peripheral blood biomarkers predict immune-related adverse events in non-small cell lung cancer patients treated with pembrolizumab: A multicenter retrospective study. | Retrospective  Multi-centre  Cohort study | Non-small cell lung cancer | III, IV | Monotherapy | Pembrolizumab | 45 | 92 | 48.9 | General irAE |

| **Author Year** | **Study Title** | **Study Design** | **Cancer Type** | **Cancer Stage (if specified)** | **ICI Type(s)** | **Specific ICI Agents** | **Patients With irAE** | **ICI Patient Population** | **Event Rate (%)** | **Type of irAE** |
| --- | --- | --- | --- | --- | --- | --- | --- | --- | --- | --- |
| Eshghi 2018 | F-18-FDG PET/CT can predict development of thyroiditis due to immunotherapy for lung cancer. | Retrospective Single-centre Cohort study | Non-small cell lung cancer | Advanced | Monotherapy | Nivolumab | 6 | 18 | 33.3 | Endocrine |
| Espi 2021 | Renal adverse effects of immune checkpoints inhibitors in clinical practice: ImmuNoTox Study. | Retrospective Single-centre Cohort study | Pan-cancer | Mostly advanced | Monotherapy | Nivolumab; Pembrolizumab; Atezolizumab; Avelumab; Durvalumab; Ipilimumab | 13 | 352 | 3.7 | Renal |
| Eun 2019 | Risk factors for immune-related adverse events associated with anti-PD-1 pembrolizumab. | Retrospective Single-centre Cohort study | Pan-cancer | Not specified | Monotherapy | Pembrolizumab | 67 | 391 | 17.1 | General irAE |
| Farha 2021 | Immune checkpoint inhibitor induced colitis: A nationwide population-based study. | Retrospective  Multi-centre  Cohort study | Pan-cancer | Not specified | Monotherapy | Nivolumab; Pembrolizumab; Atezolizumab; Ipilimumab | 470 | 13010 | 3.6 | Gastrointestinal |
| Frelau 2021 | Increased thyroid uptake on 18F-FDG PET/CT is associated with the development of permanent hypothyroidism in stage IV melanoma patients treated with anti-PD-1 antibodies. | Retrospective Single-centre Cohort study | Melanoma | IV, M1a-d | Combination & monotherapy | Nivolumab; Pembrolizumab; Combination Ipilimumab + Nivolumab; Combination Ipilimumab + Pembrolizumab | 4 | 29 | 13.8 | Endocrine |
| Friedlander 2018 | A whole-blood RNA transcript-based gene signature is associated with the development of CTLA-4 blockade-related diarrhea in patients with advanced melanoma treated with the checkpoint inhibitor tremelimumab. | Prospective  Single-centre Experimental study | Melanoma | III, IV M1a-d | Monotherapy | Tremelimumab | 77 | 360 | 21.4 | Gastrointestinal (grade specific) |
| Fujimoto 2021 | Association between pretreatment neutrophil-to-lymphocyte ratio and immune-related adverse events due to immune checkpoint inhibitors in patients with non-small cell lung cancer. | Retrospective Single-centre Cohort study | Non-small cell lung cancer | I to IV | Monotherapy | Nivolumab; Pembrolizumab; Atezolizumab | 45 | 115 | 39.1 | General irAE |
| Fujimura 2018 | Serum levels of soluble CD163 and CXCL5 may be predictive markers for immune-related adverse events in patients with advanced melanoma treated with nivolumab: A pilot study. | Prospective  Multi-centre  Pilot study | Melanoma | III, IV | Monotherapy | Nivolumab | 22 | 46 | 47.8 | General irAE |
| Fujisawa 2017 | Fluctuations in routine blood count might signal severe immune-related adverse events in melanoma patients treated with nivolumab. | Retrospective  Multi-centre  Cohort study | Melanoma | IV | Monotherapy | Nivolumab | 38 | 101 | 37.6 | General irAE |
| Fukihara 2019 | Prognostic impact and risk factors of immune-related pneumonitis in patients with non-small-cell lung cancer who received programmed death 1 inhibitors. | Retrospective  Multi-centre  Cohort study | Non-small cell lung cancer | Advanced or recurrent | Monotherapy | Nivolumab; Pembrolizumab | 27 | 170 | 15.9 | Pulmonary |

| **Author Year** | **Study Title** | **Study Design** | **Cancer Type** | **Cancer Stage (if specified)** | **ICI Type(s)** | **Specific ICI Agents** | **Patients With irAE** | **ICI Patient Population** | **Event Rate (%)** | **Type of irAE** |
| --- | --- | --- | --- | --- | --- | --- | --- | --- | --- | --- |
| Fukushima 2020 | Impact of sarcopenia on the efficacy of pembrolizumab in patients with advanced urothelial carcinoma: A preliminary report. | Retrospective Single-centre Cohort study | Urothelial carcinoma | Advanced | Monotherapy | Pembrolizumab | 7 | 28 | 25.0 | General irAE |
|  |  |  |  |  |  |  | 4 |  | 14.3 | High grade irAE |
| Gambichler 2018 | Baseline laboratory parameters predicting clinical outcome in melanoma patients treated with ipilimumab: A single-centre analysis. | Retrospective Single-centre Cohort study | Melanoma | IIIc, IV M1a-d | Monotherapy | Ipilimumab | 23 | 52 | 44.2 | General irAE |
| Garcia-Carro 2021 | Acute kidney injury as a risk factor for mortality in oncological patients receiving check-point inhibitors. | Retrospective Single-centre Cohort study | Pan-cancer | Not specified | Combination & monotherapy | Anti-PD-1; Anti-PD-L1; Anti-CTLA-4; Combination Anti-PD1/L1 + Anti-CTLA-4; Other ICI (not specified) | 118 | 759 | 15.5 | Renal |
| Giannicola 2019 | Early blood rise in auto-antibodies to nuclear and smooth muscle antigens is predictive of prolonged survival and autoimmunity in metastatic-non-small cell lung cancer patients treated with PD-1 immune-check point blockade by nivolumab. | Retrospective  Multi-centre  Cohort study | Non-small cell lung cancer | Metastatic | Monotherapy | Nivolumab | 43 | 92 | 46.7 | General irAE |
| Giordan 2021 | Impact of antibiotics and proton pump inhibitors on efficacy and tolerance of anti-PD-1 immune checkpoint inhibitors. | Retrospective Single-centre Cohort study | Melanoma; Non-small cell lung cancer; Renal cell carcinoma; Head and neck cancer; (Pan-cancer approach) | III, IV | Combination & monotherapy | Combination Anti-PD-1 + Anti-CTLA-4; Nivolumab; Pembrolizumab | 78 | 212 | 36.8 | General irAE |
| Gomes 2021 | A prospective cohort study on the safety of checkpoint inhibitors in older cancer patients - the ELDERS study. | Prospective  Multi-centre Observational study | Melanoma; Non-small cell lung cancer; (Pan-cancer approach) | III, IV M1a-c | Monotherapy | Nivolumab; Pembrolizumab; Atezolizumab; Durvalumab; Ipilimumab | 78 | 140 | 55.7 | General irAE |
|  |  |  |  |  |  |  | 22 |  | 15.7 | High grade irAE |
| Gong 2021 | Pericardial disease in patients treated with immune checkpoint inhibitors. | Retrospective Single-centre  Case-control study | Pan-cancer | Not specified | Combination & monotherapy | Anti-PD-1; Anti-PD-L1; Anti-CTLA-4; Combination Anti-PD-1 + Anti-CTLA-4 | 42 | 2842 | 1.5 | Cardiac |
| Gowen 2018 | Baseline antibody profiles predict toxicity in melanoma patients treated with immune checkpoint inhibitors. | Prospective  Single-centre Experimental study | Melanoma | Metastatic | Combination & monotherapy | Anti-PD-1; Anti-CTLA-4; Combination Anti-PD-1 + Anti-CTLA-4 | 63 | 75 | 84.0 | General irAE |
| Grover 2020 (1) | Safety of immune checkpoint inhibitors in patients with pre-existing inflammatory bowel disease and microscopic colitis. | Retrospective Single-centre Cohort study | Pan-cancer | Not specified | Combination & monotherapy | Anti-PD-1; Anti-PD-L1; Ipilimumab; Combination Ipilimumab + Nivolumab | 7 | 548 | 1.3 | Gastrointestinal |

| **Author Year** | **Study Title** | **Study Design** | **Cancer Type** | **Cancer Stage (if specified)** | **ICI Type(s)** | **Specific ICI Agents** | **Patients With irAE** | **ICI Patient Population** | **Event Rate (%)** | **Type of irAE** |
| --- | --- | --- | --- | --- | --- | --- | --- | --- | --- | --- |
| Grover 2020 (2) | Vitamin D intake is associated with decreased risk of immune checkpoint inhibitor-induced colitis. | Retrospective Single-centre Cohort study | Melanoma | III, IV | Combination & monotherapy | Nivolumab; Pembrolizumab; Ipilimumab; Combination Ipilimumab + Nivolumab | 37 | 213 | 17.4 | Gastrointestinal |
| Gulave 2021 | High body mass index and pre-existing autoimmune disease are associated with an increased risk of immune-related adverse events in cancer patients treated with PD-(L)1 inhibitors across different solid tumors. | Retrospective Clinical trial subset analysis | Pan-cancer | Not specified | Monotherapy | Nivolumab; Pembrolizumab; Atezolizumab; Durvalumab | 983 | 4265 | 23.0 | General irAE |
|  |  |  |  |  |  |  | 246 |  | 5.8 | High grade irAE |
| Gupta 2021 | Acute kidney injury in patients treated with immune checkpoint inhibitors. | Retrospective  Multi-centre  Cohort study | Pan-cancer | Not specified | Combination & monotherapy | Anti-PD-1; Anti-PD-L1; Anti-CTLA-4; Combination Anti-PD1/L1 + Anti-CTLA-4 | 429 | 858 | 50.0 | Renal |
| Guzman-Prado 2021 (1) | Body mass index and immune-related adverse events in patients on immune checkpoint inhibitor therapies: A systematic review and meta-analysis. | Systematic review & meta-analysis | Pan-cancer | Not specified | Combination & monotherapy | Anti-PD-1; Anti-PD-L1; Anti-CTLA-4; Combination Anti-PD1/L1 + Anti-CTLA-4 | 622 | 1937 | 32.1 | General irAE |
| Guzman-Prado 2021 (2) | Sarcopenia and the risk of adverse events in patients treated with immune checkpoint inhibitors: A systematic review. | Systematic review | Pan-cancer | Not specified | Combination & monotherapy | Anti-PD-1; Anti-PD-L1; Combination Anti-PD1/L1 + Anti-CTLA-4; Nivolumab; Pembrolizumab; Ipilimumab | Not specified | 793 | Not assessable | General irAE |
| Haddad 2021 | Immune checkpoint inhibitor-related thrombocytopenia: Incidence, risk factors and effect on survival. | Retrospective Single-centre Cohort study | Pan-cancer | Advanced | Combination & monotherapy | Nivolumab; Pembrolizumab; Atezolizumab; Ipilimumab; Combination Ipilimumab + Nivolumab | 18 | 1038 | 1.7 | High grade irAE |
| Haik 2021 | The impact of sarcopenia on the efficacy and safety of immune checkpoint inhibitors in patients with solid tumours. | Retrospective Single-centre Cohort study | Pan-cancer | Not specified | Combination & monotherapy | Anti-PD-1; Anti-PD-L1; Anti-CTLA-4; Combination Anti-PD1/L1 + Anti-CTLA-4 | 17 | 261 | 6.5 | General irAE |
| Hakozaki 2020 (1) | Polypharmacy as a prognostic factor in older patients with advanced non-small-cell lung cancer treated with anti-PD-1/PD-L1 antibody-based immunotherapy. | Prospective  Multi-centre  Cohort study | Non-small cell lung cancer | IIIa-b, IVa-b | Monotherapy | Nivolumab; Pembrolizumab; Atezolizumab | 16 | 70 | 22.9 | High grade irAE |
| Hakozaki 2020 (2) | The gut microbiome associates with immune checkpoint inhibition outcomes in patients with advanced non-small cell lung cancer. | Retrospective  Multi-centre  Cohort study | Non-small cell lung cancer | III, IVa-b | Monotherapy | Nivolumab; Pembrolizumab; Atezolizumab | 33 | 157 | 21.0 | High grade irAE |

| **Author Year** | **Study Title** | **Study Design** | **Cancer Type** | **Cancer Stage (if specified)** | **ICI Type(s)** | **Specific ICI Agents** | **Patients With irAE** | **ICI Patient Population** | **Event Rate (%)** | **Type of irAE** |
| --- | --- | --- | --- | --- | --- | --- | --- | --- | --- | --- |
| Han 2021 | Cutaneous adverse events associated with immune checkpoint blockade: A systematic review and meta-analysis. | Systematic review & meta-analysis | Pan-cancer | Not specified | Monotherapy | Nivolumab; Pembrolizumab; Atezolizumab; Avelumab; Durvalumab; Ipilimumab; Tremelimumab; (non-ICI cohort included; analysis performed separately) | 3579 | 10101 | 35.4 | Skin |
| HasanAli 2019 | Human leukocyte antigen variation is associated with adverse events of checkpoint inhibitors. | Prospective  Single-centre Observational study | Melanoma; Non-small cell lung cancer (Pan cancer approach) | Mostly IV | Combination & monotherapy | Nivolumab; Pembrolizumab; Atezolizumab; Ipilimumab; Combination Ipilimumab + Nivolumab | 59 | 102 | 57.8 | General irAE |
| Hasan Ali 2020 | BP180-specific IgG is associated with skin adverse events, therapy response, and overall survival in non-small cell lung cancer patients treated with checkpoint inhibitors. | Prospective  Single-centre  Cohort study | Non-small cell lung cancer | Not specified | Monotherapy | Nivolumab; Pembrolizumab; Atezolizumab | 25 | 40 | 62.5 | General irAE |
|  |  |  |  |  |  |  | 16 |  | 40.0 | Skin |
| He 2021 | Pan-cancer analysis reveals alternative splicing characteristics associated with immune-related adverse events elicited by checkpoint immunotherapy. | Retrospective Experimental study | Pan-cancer | Not specified | Monotherapy | Nivolumab; Pembrolizumab; Cemiplimab; Atezolizumab; Avelumab; Durvalumab | Not specified | 6906 | Not assessable | General irAE |
| Heidelberger 2017 | Sarcopenic overweight is associated with early acute limiting toxicity of anti-PD1 checkpoint inhibitors in melanoma patients. | Retrospective Single-centre Cohort study | Melanoma | Not specified | Monotherapy | Nivolumab; Pembrolizumab | 11 | 77 | 14.3 | General irAE |
| Heilbroner 2021 | Predicting cardiac adverse events in patients receiving immune checkpoint inhibitors: A machine learning approach. | Retrospective  Multi-centre  Cohort study | Melanoma; Non-small cell lung cancer; Renal cell carcinoma | All stages | Monotherapy | Nivolumab; Pembrolizumab; Atezolizumab; Avelumab; Durvalumab | 418 | 4960 | 8.4 | Cardiac |
| Hirsch 2020 | The impact of body composition parameters on severe toxicity of nivolumab. | Prospective  Single-centre  Cohort study | Pan-cancer | Not specified | Monotherapy | Nivolumab | 22 | 92 | 23.9 | High grade irAE |
| Hribernik | Quantitative imaging biomarkers of immune-related adverse events in immune-checkpoint blockade-treated metastatic melanoma patients: A pilot study. | Retrospective  Multi-centre  Cohort study | Melanoma | Metastatic | Monotherapy | Anti-PD-1; Anti-CTLA-4 | 13 | 58 | 22.4 | General irAE |
| Husain 2021 | Inflammatory markers in autoimmunity induced by checkpoint inhibitors. | Prospective  Single-centre  Cohort study | Melanoma | Metastatic | Combination & monotherapy | Combination Anti-PD-1 + Anti-CTLA-4; Nivolumab; Pembrolizumab; Ipilimumab | 13 | 16 | 81.3 | General irAE |

| **Author Year** | **Study Title** | **Study Design** | **Cancer Type** | **Cancer Stage (if specified)** | **ICI Type(s)** | **Specific ICI Agents** | **Patients With irAE** | **ICI Patient Population** | **Event Rate (%)** | **Type of irAE** |
| --- | --- | --- | --- | --- | --- | --- | --- | --- | --- | --- |
| Iacovelli 2017 | Renal toxicity in patients treated with anti-PD-1 targeted agents for solid tumors. | Systematic review & meta-analysis | Pan-cancer | III, IV | Monotherapy | Anti-PD-1 | 48 | 3455 | 1.4 | Renal (grade specific) |
| Iafolla 2021 | Predicting toxicity and response to pembrolizumab through germline genomic HLA class 1 analysis. | Prospective  Single-centre Clinical trial subset analysis | Pan-cancer | Advanced | Monotherapy | Pembrolizumab | 23 | 101 | 22.8 | General irAE |
| Ikeda 2020 | A phase 2 study of atezolizumab for pretreated NSCLC with idiopathic interstitial pneumonitis. | Prospective  Multi-centre  Clinical trial subset analysis | Non-small cell lung cancer | IIIa-c, IVa-b, recurrent | Monotherapy | Atezolizumab | 5 | 17 | 29.4 | Pulmonary |
| Ikeda 2021 | Prognostic impact of immune-related adverse events in metastatic renal cell carcinoma treated with nivolumab plus ipilimumab. | Retrospective  Multi-centre  Cohort study | Renal cell carcinoma | Metastatic | Combination only | Combination Ipilimumab + Nivolumab | 33 | 46 | 71.7 | General irAE |
| Iravani 2020 | FDG PET/CT for tumoral and systemic immune response monitoring of advanced melanoma during first-line combination ipilimumab and nivolumab treatment. | Retrospective Single-centre Cohort study | Melanoma | IIIb, IVa-d | Combination only | Combination Ipilimumab + Nivolumab | 20 | 31 | 64.5 | General irAE |
| Ishida 2018 | Killer immunoglobulin-like receptor genotype did not correlate with response to anti-PD-1 antibody treatment in a Japanese cohort. | Prospective  Multi-centre  Cohort study | Melanoma | Not specified | Monotherapy | Nivolumab | 35 | 112 | 31.3 | General irAE |
| Ishihara 2019 | Association between immune-related adverse events and prognosis in patients with metastatic renal cell carcinoma treated with nivolumab. | Retrospective  Multi-centre  Cohort study | Renal cell carcinoma | Metastatic | Monotherapy | Nivolumab | 23 | 47 | 48.9 | General irAE |
|  |  |  |  |  |  |  | 10 |  | 21.3 | High grade irAE |
| Isik 2021 | Biomarkers, clinical features, and rechallenge for immune checkpoint inhibitor renal immune-related adverse events. | Retrospective Single-centre Cohort study | Pan-cancer | Not specified | Combination & monotherapy | Combination Anti-PD1/L1 + Anti-CTLA-4; Nivolumab; Pembrolizumab; Atezolizumab; Avelumab; Durvalumab; Ipilimumab | 37 | 2143 | 1.7 | Renal |
| Isono 2021 | Outcome and risk factor of immune-related adverse events and pneumonitis in patients with advanced or postoperative recurrent non-small cell lung cancer treated with immune checkpoint inhibitors. | Retrospective Single-centre Cohort study | Non-small cell lung cancer | III, IV | Monotherapy | Nivolumab; Pembrolizumab; Atezolizumab | 85 | 180 | 47.2 | General irAE |
|  |  |  |  |  |  |  | 27 |  | 15.0 | Pulmonary |
| Jing 2020 | Multi-omics prediction of immune-related adverse events during checkpoint immunotherapy. | Retrospective  Multi-centre Experimental study | Pan-cancer | Not specified | Monotherapy | Nivolumab; Pembrolizumab; Cemiplimab; Atezolizumab; Avelumab; Durvalumab | 3706 | 18706 | 19.8 | General irAE |

| **Author Year** | **Study Title** | **Study Design** | **Cancer Type** | **Cancer Stage (if specified)** | **ICI Type(s)** | **Specific ICI Agents** | **Patients With irAE** | **ICI Patient Population** | **Event Rate (%)** | **Type of irAE** |
| --- | --- | --- | --- | --- | --- | --- | --- | --- | --- | --- |
| Joshi 2020 | Immunotherapy outcomes in advanced melanoma in relation to age. | Retrospective Single-centre Cohort study | Melanoma | IV | Combination & monotherapy | Nivolumab; Pembrolizumab; Ipilimumab; Combination Ipilimumab + Nivolumab | 32 | 50 | 64.0 | General irAE |
| Kahler 2018 | Ipilimumab in metastatic melanoma patients with pre-existing autoimmune disorders. | Retrospective  Multi-centre  Cohort study | Melanoma | Metastatic | Monotherapy | Ipilimumab | 12 | 41 | 29.3 | General irAE |
| Kalinich 2021 | Prediction of severe immune-related adverse events requiring hospital admission in patients on immune checkpoint inhibitors: Study of a population level insurance claims database from the USA. | Retrospective  Multi-centre  Cohort study | Pan-cancer | Not specified | Combination & monotherapy | Nivolumab; Pembrolizumab; Atezolizumab; Avelumab; Ipilimumab; Combination Ipilimumab + Nivolumab; Combination Ipilimumab + Pembrolizumab | 504 | 14378 | 3.5 | General irAE |
| Kanai 2018 | Efficacy and safety of nivolumab in non-small cell lung cancer with preexisting interstitial lung disease. | Retrospective  Multi-centre  Cohort study | Non-small cell lung cancer | Advanced or recurrent | Monotherapy | Nivolumab | 30 | 216 | 13.9 | Pulmonary |
| Kano 2021 | Association between skeletal muscle loss and the response to nivolumab immunotherapy in advanced gastric cancer patients. | Retrospective Single-centre Cohort study | Gastric cancer | Advanced | Monotherapy | Nivolumab | 8 | 31 | 25.8 | General irAE |
| Kazama 2021 | Prognostic impact of immune-related adverse events on patients with and without cardiovascular disease: A retrospective review. | Retrospective Single-centre Cohort study | Pan-cancer | Not specified | Combination & monotherapy | Nivolumab; Pembrolizumab; Atezolizumab; Durvalumab; Ipilimumab; Combination Ipilimumab + Nivolumab | 14 | 409 | 3.4 | Cardiac |
| Kehl 2019 | Pre-existing autoimmune disease and the risk of immune-related adverse events among patients receiving checkpoint inhibitors for cancer. | Retrospective  Multi-centre  Cohort study | Pan-cancer | Not specified | Combination & monotherapy | Nivolumab; Pembrolizumab; Atezolizumab; Ipilimumab; Combination Ipilimumab + Nivolumab | 760 | 4438 | 17.1 | General irAE |
| Khan 2019 | Immune dysregulation in cancer patients developing immune-related adverse events. | Prospective  Single-centre Experimental study | Pan-cancer | Not specified | Combination & monotherapy | Anti-PD-1; Anti-PD-L1; Anti-CTLA-4; Combination Anti-PD-1 + Anti-CTLA-4 | 24 | 65 | 36.9 | General irAE |
| Khoja 2017 | Tumour- and class-specific patterns of immune-related adverse events of immune checkpoint inhibitors: A systematic review. | Systematic review | Pan-cancer | Not specified | Combination & monotherapy | Anti-PD-1; Anti-PD-L1; Anti-CTLA-4; Combination Anti-PD-1 + Anti-CTLA-4 | Not specified | 6938 | Not assessable | General irAE |

| **Author Year** | **Study Title** | **Study Design** | **Cancer Type** | **Cancer Stage (if specified)** | **ICI Type(s)** | **Specific ICI Agents** | **Patients With irAE** | **ICI Patient Population** | **Event Rate (%)** | **Type of irAE** |
| --- | --- | --- | --- | --- | --- | --- | --- | --- | --- | --- |
| Khunger 2021 | Multimarker scores of Th1 and Th2 immune cellular profiles in peripheral blood predict response and immune related toxicity with CTLA4 blockade and IFNalpha in melanoma. | Prospective  Single-centre Experimental study | Melanoma | IIIb-c, IV | Combination only | Combination Ipilimumab + Nivolumab | 14 | 30 | 46.7 | General irAE |
| Kichenadasse 2020 (1) | Multiorgan immune-related adverse events during treatment with atezolizumab. | Retrospective Clinical trial subset analysis | Non-small cell lung cancer | Not specified | Monotherapy | Atezolizumab | 424 | 1548 | 27.4 | General irAE |
| Kichenadasse 2020 (2) | Association between body mass index and overall survival with immune checkpoint inhibitor therapy for advanced non-small cell lung cancer. | Retrospective  Multi-centre  Clinical trial subset analysis | Non-small cell lung cancer | Advanced | Monotherapy | Atezolizumab; (non-ICI cohort included; analysis performed separately) | 390 | 1434 | 27.2 | General irAE |
| Kim 2020 | Immune-related adverse events are clustered into distinct subtypes by T-cell profiling before and early after anti-PD-1 treatment. | Prospective  Single-centre Experimental study | Non-small cell lung cancer; Refractory thymic epithelial tumour | Metastatic or refractory | Monotherapy | Nivolumab; Pembrolizumab | 45 | 91 | 49.5 | General irAE |
|  |  |  |  |  |  |  | 13 |  | 14.3 | High grade irAE |
| Kim 2021 (1) | Factors associated with thyroid-related adverse events in patients receiving PD-1 or PD-L1 inhibitors using machine learning models. | Retrospective Single-centre Cohort study | Pan-cancer | Not specified | Monotherapy | Nivolumab; Pembrolizumab; Atezolizumab | 23 | 187 | 12.3 | Endocrine |
| Kim 2021 (2) | Distinct immunophenotypes of T cells in bronchoalveolar lavage fluid from leukemia patients with immune checkpoint inhibitors-related pulmonary complications. | Prospective  Single-centre Experimental study | Acute myeloid leukemia (AML) and myelodysplastic syndrome (MDS) | Not applicable | Combination & monotherapy | Nivolumab; Avelumab; Ipilimumab; Combination Ipilimumab + Nivolumab | 3 | 7 | 42.9 | Pulmonary |
| Kimbara 2018 | Association of antithyroglobulin antibodies with the development of thyroid dysfunction induced by nivolumab. | Retrospective Single-centre Cohort study | Pan-cancer | Not specified | Monotherapy | Nivolumab | 168 | 256 | 65.6 | Endocrine |
| Kitagataya 2020 | Prevalence, clinical course, and predictive factors of immune checkpoint inhibitor monotherapy-associated hepatitis in Japan. | Retrospective Single-centre Cohort study | Pan-cancer | Not specified | Monotherapy | Nivolumab; Pembrolizumab; Atezolizumab; Avelumab | 17 | 202 | 8.4 | Gastrointestinal (grade specific) |
| Kobayashi 2018 | Patients with antithyroid antibodies are prone to develop destructive thyroiditis by nivolumab: A prospective study. | Prospective  Single-centre  Cohort study | Melanoma; Non-small cell lung cancer; Renal cell carcinoma; Hodgkin's lymphoma | Advanced or metastatic | Monotherapy | Nivolumab | 4 | 66 | 6.1 | Endocrine |
| Kobayashi 2021 | Anti-pituitary antibodies and susceptible human leukocyte antigen alleles as predictive biomarkers for pituitary dysfunction induced by immune checkpoint inhibitors. | Retrospective Single-centre  Case-control study | Pan-cancer | Not specified | Combination & monotherapy | Nivolumab; Pembrolizumab; Atezolizumab; Ipilimumab; Combination Ipilimumab + Nivolumab | 22 | 62 | 35.5 | Endocrine |

| **Author Year** | **Study Title** | **Study Design** | **Cancer Type** | **Cancer Stage (if specified)** | **ICI Type(s)** | **Specific ICI Agents** | **Patients With irAE** | **ICI Patient Population** | **Event Rate (%)** | **Type of irAE** |
| --- | --- | --- | --- | --- | --- | --- | --- | --- | --- | --- |
| Koks 2021 | Immune checkpoint inhibitor-associated acute kidney injury and mortality: An observational study. | Retrospective Single-centre Observational study | Pan-cancer | Not specified | Combination & monotherapy | Nivolumab; Pembrolizumab; Atezolizumab; Durvalumab; Ipilimumab; Combination Ipilimumab + Nivolumab; Tremelimumab | 96 | 676 | 14.2 | Renal |
| Komiya 2019 | Discontinuation due to immune-related adverse events is a possible predictive factor for immune checkpoint inhibitors in patients with non-small cell lung cancer. | Retrospective Single-centre Cohort study | Non-small cell lung cancer | III, IV, recurrent | Monotherapy | Nivolumab; Pembrolizumab | 18 | 61 | 29.5 | General irAE |
| Kono 2021 | Association between immune-related adverse events and the prognosis of patients with advanced gastric cancer treated with nivolumab. | Retrospective Single-centre Cohort study | Gastric cancer | Advanced or recurrent unresectable | Monotherapy | Nivolumab | 13 | 52 | 25.0 | General irAE |
| Kotwal 2019 | Immune checkpoint inhibitors: An emerging cause of insulin-dependent diabetes. | Retrospective Single-centre Cohort study | Pan-cancer | Not specified | Monotherapy | Nivolumab; Pembrolizumab; Ipilimumab | 21 | 1444 | 1.5 | Endocrine |
| Kotwal 2020 | PD-L1 inhibitor-induced thyroiditis is associated with better overall survival in cancer patients. | Retrospective Single-centre Cohort study | Pan-cancer | Not specified | Monotherapy | Nivolumab; Atezolizumab; Avelumab | 19 | 91 | 20.9 | Endocrine |
| Koyama 2019 | Correlation between thyroid transcription factor-1 expression, immune-related thyroid dysfunction, and efficacy of anti-programmed cell death protein-1 treatment in non-small cell lung cancer. | Retrospective Single-centre Cohort study | Non-small cell lung cancer | Not specified | Monotherapy | Nivolumab; Pembrolizumab | 19 | 132 | 14.4 | Endocrine |
| Krishnan 2020 | A retrospective analysis of eosinophilia as a predictive marker of response and toxicity to cancer immunotherapy. | Retrospective Single-centre Cohort study | Pan-cancer | II, III, IV | Combination & monotherapy | Nivolumab; Pembrolizumab; Atezolizumab; Durvalumab; Ipilimumab; Combination Ipilimumab + Nivolumab | 66 | 146 | 45.2 | General irAE |
|  |  |  |  |  |  |  | 15 |  | 10.3 | High grade irAE |
| Ksienski 2020 | Association of age with differences in immune related adverse events and survival of patients with advanced nonsmall cell lung cancer receiving pembrolizumab or nivolumab. | Retrospective  Multi-centre  Cohort study | Non-small cell lung cancer | Advanced | Monotherapy | Nivolumab; Pembrolizumab | 121 | 527 | 23.0 | General irAE |
| Ksienski 2021 | Prognostic significance of the neutrophil-to-lymphocyte ratio and platelet-to-lymphocyte ratio for advanced non-small cell lung cancer patients with high PD-L1 tumor expression receiving pembrolizumab. | Retrospective  Multi-centre  Cohort study | Non-small cell lung cancer | IV | Monotherapy | Pembrolizumab | 89 | 220 | 40.5 | General irAE |

| **Author Year** | **Study Title** | **Study Design** | **Cancer Type** | **Cancer Stage (if specified)** | **ICI Type(s)** | **Specific ICI Agents** | **Patients With irAE** | **ICI Patient Population** | **Event Rate (%)** | **Type of irAE** |
| --- | --- | --- | --- | --- | --- | --- | --- | --- | --- | --- |
| Kurimoto 2020 | Predictive and sensitive biomarkers for thyroid dysfunctions during treatment with immune-checkpoint inhibitors. | Prospective  Single-centre Observational study | Melanoma; Non-small cell lung cancer; Renal cell carcinoma; Upper gastrointestinal cancer; Genitourinary cancer | Not specified | Combination & monotherapy | Nivolumab; Pembrolizumab; Ipilimumab; Combination Ipilimumab + Nivolumab | 13 | 26 | 50.0 | Endocrine |
| Kurzhals 2021 | Serum troponin T concentrations are frequently elevated in advanced skin cancer patients prior to immune checkpoint inhibitor therapy: Experience from a single tertiary referral center. | Retrospective Single-centre Cohort study | Melanoma; Non-melanoma skin cancer | Adjuvant, mostly IV | Monotherapy | Nivolumab; Pembrolizumab; Cemiplimab; Avelumab | 1 | 121 | 0.8 | Cardiac |
| L'Orphelin 2021 | Severe late-onset grade III-IV adverse events under immunotherapy: A retrospective study of 79 cases. | Retrospective  Multi-centre  Cohort study | Melanoma | III, IV | Combination & monotherapy | Nivolumab; Pembrolizumab; Combination Ipilimumab + Nivolumab | 79 | 1862 | 4.2 | General irAE |
| Leiter 2020 | Characterization of immune checkpoint inhibitor-mediated hyperglycemia: Beyond insulin-dependent diabetes. | Retrospective Single-centre Cohort study | Pan-cancer | IV | Monotherapy | Nivolumab; Pembrolizumab; Atezolizumab; Ipilimumab; Tremelimumab | 10 | 385 | 2.6 | Endocrine |
| Leiter 2021 | Metabolic disease and adverse events from immune checkpoint inhibitors. | Retrospective Single-centre Observational study | Pan-cancer | Clinically localized, regionally advanced, distant metastasis, other (haematologic) | Monotherapy | Nivolumab; Pembrolizumab; Atezolizumab; Ipilimumab; Tremelimumab | 111 | 374 | 29.7 | High grade irAE |
| Leonardi 2018 | Safety of programmed death-1 pathway inhibitors among patients with non-small-cell lung cancer and preexisting autoimmune disorders. | Retrospective  Multi-centre  Cohort study | Non-small cell lung cancer | IIIb, IV | Monotherapy | Nivolumab; Pembrolizumab; Atezolizumab | 21 | 56 | 37.5 | General irAE |
| Les 2021 | Association of immune-related adverse events induced by nivolumab with a battery of autoantibodies. | Retrospective Single-centre Cohort study | Pan-cancer | IV | Monotherapy | Nivolumab | 26 | 69 | 37.7 | General irAE |
| Li 2020 | Brief report: Inhaled corticosteroid use and the risk of checkpoint inhibitor pneumonitis in patients with advanced cancer. | Retrospective Single-centre Cohort study | Pan-cancer | Advanced | Combination & monotherapy | Nivolumab; Pembrolizumab; Ipilimumab; Combination Ipilimumab + Nivolumab; Other ICI (not specified) | 30 | 837 | 3.6 | Pulmonary (grade specific) |
| Li 2021 (1) | A digital single-molecule nanopillar SERS platform for predicting and monitoring immune toxicities in immunotherapy. | Prospective  Single-centre Experimental study | Melanoma | Not specified | Monotherapy | Pembrolizumab; Ipilimumab; Other ICI (not specified) | 5 | 10 | 50.0 | High grade irAE |

| **Author Year** | **Study Title** | **Study Design** | **Cancer Type** | **Cancer Stage (if specified)** | **ICI Type(s)** | **Specific ICI Agents** | **Patients With irAE** | **ICI Patient Population** | **Event Rate (%)** | **Type of irAE** |
| --- | --- | --- | --- | --- | --- | --- | --- | --- | --- | --- |
| Li 2021 (2) | Prognostic impact of sarcopenia on clinical outcomes in malignancies treated with immune checkpoint inhibitors: A systematic review and meta-analysis. | Systematic review & meta-analysis | Pan-cancer | Not specified | Combination & monotherapy | Anti-PD-1; Anti-PD-L1; Nivolumab; Pembrolizumab; Atezolizumab; Avelumab; Durvalumab; Ipilimumab; Combination Ipilimumab + Nivolumab | Not specified | 1763 | Not assessable | General irAE & high grade irAE |
| Li 2021 (3) | Prognostic impact of sarcopenia on immune-related adverse events in malignancies received immune checkpoint inhibitors: A systematic review and meta-analysis. | Systematic review & meta-analysis | Pan-cancer | Not specified | Monotherapy | Anti-PD-1; Anti-PD-L1; Anti-CTLA-4 | Not specified | 519 | Not assessable | General irAE & high grade irAE |
| Lim 2019 | Circulating cytokines predict immune-related toxicity in melanoma patients receiving anti-PD-1-based immunotherapy. | Prospective  Multi-centre Experimental study | Melanoma | III, IV M1a-c | Combination only | Discovery cohort 2: Combination Ipilimumab + Nivolumab; Combination Ipilimumab + Pembrolizumab | 24 | 58 | 41.4 | General irAE |
| Lin 2021 | Peripheral blood biomarkers for early diagnosis, severity, and prognosis of checkpoint inhibitor-related pneumonitis in patients with lung cancer. | Retrospective Single-centre  Case-control study | Lung cancer | III, IV | Monotherapy | Anti-PD-1; Anti-PD-L1 | 87 | 848 | 10.3 | Pulmonary |
| Lisberg 2018 | Treatment-related adverse events predict improved clinical outcome in NSCLC patients on KEYNOTE-001 at a single center. | Retrospective Single-centre Cohort study | Non-small cell lung cancer | Not specified | Monotherapy | Pembrolizumab | 6 | 97 | 6.2 | General irAE |
| Liu 2018 | Intestinal microbiota to predict risk for immune-related diarrhea in patients with lung cancer patients. | Prospective  Single-centre  Cohort study | Non-small cell lung cancer; Small cell lung cancer | Not specified | Monotherapy | Nivolumab; Pembrolizumab | 8 | 26 | 30.8 | Gastrointestinal |
| Liu 2021 | Peripheral blood markers associated with immune-related adverse effects in patients who had advanced non-small cell lung cancer treated with PD-1 inhibitors. | Prospective  Single-centre Observational study | Non-small cell lung cancer | III, IV | Monotherapy | Nivolumab; Pembrolizumab | 57 | 150 | 38.0 | General irAE |
|  |  |  |  |  |  |  | 15 |  | 10.0 | High grade irAE |
| Lu 2021 | Immune-related adverse events predict responses to PD-1 blockade immunotherapy in hepatocellular carcinoma. | Retrospective Single-centre Cohort study | Pan-cancer | Not specified | Monotherapy | Nivolumab; Pembrolizumab; Sintilimab; Toripalimab | 21 | 101 | 20.8 | General irAE |
| Ma 2019 | The impact of high-dose glucocorticoids on the outcome of immune-checkpoint inhibitor-related thyroid disorders. | Retrospective Single-centre Cohort study | Pan-cancer | Not specified | Combination & monotherapy | Nivolumab; Pembrolizumab; Combination Ipilimumab + Nivolumab | 53 | 151 | 35.1 | Endocrine |

| **Author Year** | **Study Title** | **Study Design** | **Cancer Type** | **Cancer Stage (if specified)** | **ICI Type(s)** | **Specific ICI Agents** | **Patients With irAE** | **ICI Patient Population** | **Event Rate (%)** | **Type of irAE** |
| --- | --- | --- | --- | --- | --- | --- | --- | --- | --- | --- |
| Ma 2021 | Characterization of outcomes in patients with advanced genitourinary malignancies treated with immune checkpoint inhibitors. | Retrospective Single-centre Cohort study | Renal cell carcinoma; Urothelial cancer | III, IV | Combination & monotherapy | Nivolumab; Pembrolizumab; Atezolizumab; Combination Ipilimumab + Nivolumab | 82 | 160 | 51.3 | General irAE |
| Maekura 2017 | Predictive factors of nivolumab-induced hypothyroidism in patients with non-small cell lung cancer. | Retrospective Single-centre Cohort study | Non-small cell lung cancer | Not specified | Monotherapy | Nivolumab | 5 | 64 | 7.8 | Endocrine |
| Mahmood 2018 | Myocarditis in patients treated with immune checkpoint inhibitors. | Retrospective  Multi-centre  Case-control study | Pan-cancer | Not specified | Combination & monotherapy | Nivolumab; Pembrolizumab; Atezolizumab; Avelumab; Durvalumab; Ipilimumab; Combination Ipilimumab + Nivolumab; Combination Ipilimumab + Pembrolizumab; Tremelimumab; Combination Tremelimumab + Durvalumab; Combination Tremelimumab + Avelumab | 35 | 140 | 25.0 | Cardiac |
| Maillet 2020 | Association between immune-related adverse events and long-term survival outcomes in patients treated with immune checkpoint inhibitors. | Retrospective Single-centre Cohort study | Pan-cancer | Not specified | Monotherapy | Anti-PD-L1; Anti-CTLA-4 | 126 | 410 | 30.7 | General irAE |
|  |  |  |  |  |  |  | 49 |  | 12.0 | High grade irAE |
| Manne 2021 | Clinical and hematological predictors of high-grade immune-related adverse events associated with immune checkpoint inhibitors. | Retrospective Single-centre Cohort study | Melanoma; Lung cancer | II, III, IV | Combination & monotherapy | Nivolumab; Pembrolizumab; Atezolizumab; Durvalumab; Ipilimumab; Combination Ipilimumab + Nivolumab | 46 | 160 | 28.8 | High grade irAE |
| Mao 2021 | Gut microbiome is associated with the clinical response to anti-PD-1 based immunotherapy in hepatobiliary cancers. | Prospective  Single-centre  Cohort study | Liver cancer | II, III, IV | Monotherapy | Anti-PD-1 | 8 | 65 | 12.3 | Gastrointestinal (grade specific) |

| **Author Year** | **Study Title** | **Study Design** | **Cancer Type** | **Cancer Stage (if specified)** | **ICI Type(s)** | **Specific ICI Agents** | **Patients With irAE** | **ICI Patient Population** | **Event Rate (%)** | **Type of irAE** |
| --- | --- | --- | --- | --- | --- | --- | --- | --- | --- | --- |
| Matsukane 2021 | Continuous monitoring of neutrophils to lymphocytes ratio for estimating the onset, severity, and subsequent prognosis of immune related adverse events. | Retrospective Single-centre Cohort study | Melanoma; Non-small cell lung cancer; Renal cell carcinoma; Head and neck cancer; (Pan-cancer approach) | Metastatic or unresectable | Monotherapy | Nivolumab; Pembrolizumab | 121 | 275 | 44.0 | General irAE |
|  |  |  |  |  |  |  | 29 |  | 10.5 | High grade irAE |
| Matsuo 2020 | Relationship between immune-related adverse events and the long-term outcomes in recurrent/metastatic head and neck squamous cell carcinoma treated with nivolumab. | Retrospective  Multi-centre  Cohort study | Head and neck squamous cell carcinoma | Not specified | Monotherapy | Nivolumab | 41 | 108 | 38.0 | General irAE |
| Matsuoka 2020 | Correlation between immune-related adverse events and prognosis in patients with various cancers treated with anti PD-1 antibody. | Retrospective Single-centre Cohort study | Pan-cancer | Not specified | Monotherapy | Nivolumab; Pembrolizumab | 115 | 280 | 41.1 | General irAE |
| McQuade 2018 | Association of body-mass index and outcomes in patients with metastatic melanoma treated with targeted therapy, immunotherapy, or chemotherapy: A retrospective, multicohort analysis. | Retrospective  Multi-centre  Clinical trial subset analysis | Melanoma | III, M1a-c | Monotherapy | Nivolumab; Pembrolizumab; Atezolizumab; (non-ICI cohort included; analysis performed separately) | 146 | 331 | 44.1 | General irAE |
| Menzies 2017 | Anti-PD-1 therapy in patients with advanced melanoma and preexisting autoimmune disorders or major toxicity with ipilimumab. | Retrospective  Multi-centre  Cohort study | Melanoma | Mostly IV M1c | Monotherapy | Nivolumab; Pembrolizumab | 15 | 119 | 12.6 | General irAE |
| Meraz-Muñoz 2020 | Acute kidney injury associated with immune checkpoint inhibitor therapy: Incidence, risk factors and outcomes. | Retrospective Single-centre Clinical trial subset analysis | Pan-cancer | Not specified | Combination & monotherapy | Nivolumab; Pembrolizumab; Ipilimumab; Combination Ipilimumab + Nivolumab | 51 | 309 | 16.5 | Renal |
| Meserve 2021 | Systematic review with meta-analysis: Safety and tolerability of immune checkpoint inhibitors in patients with pre-existing inflammatory bowel diseases. | Systematic review & meta-analysis | Pan-cancer | Not specified | Combination & monotherapy | Anti-PD-1; Anti-PD-L1; Anti-CTLA-4; Combination Anti-PD1/L1 + Anti-CTLA-4 | 77 | 193 | 39.9 | Gastrointestinal |
| Metro 2020 | Efficacy of pembrolizumab monotherapy in patients with or without brain metastases from advanced non-small cell lung cancer with a PD-L1 expression >=50%. | Retrospective  Multi-centre  Cohort study | Non-small cell lung cancer | IV | Monotherapy | Pembrolizumab | 29 | 282 | 10.3 | General irAE |
| Mikami 2021 | Neuroimmunological adverse events associated with immune checkpoint inhibitor: A retrospective, pharmacovigilance study using FAERS database. | Retrospective Observational pharmacovigilance study | Pan-cancer | Not specified | Combination & monotherapy | Combination Anti-PD1/L1 + Anti-CTLA-4; Nivolumab; Pembrolizumab; Atezolizumab; Avelumab; Durvalumab; Ipilimumab; Tremelimumab | 3619 | Not specified | Not assessable | Neurological |
| **Author Year** | **Study Title** | **Study Design** | **Cancer Type** | **Cancer Stage (if specified)** | **ICI Type(s)** | **Specific ICI Agents** | **Patients With irAE** | **ICI Patient Population** | **Event Rate (%)** | **Type of irAE** |
| Mizuno 2020 | Real world data of liver injury induced by immune checkpoint inhibitors in Japanese patients with advanced malignancies. | Retrospective  Multi-centre  Cohort study | Pan-cancer | Advanced | Combination & monotherapy | Nivolumab; Pembrolizumab; Atezolizumab; Durvalumab; Ipilimumab; Combination Ipilimumab + Nivolumab | 29 | 546 | 5.3 | Gastrointestinal (grade specific) |
| Muchnik 2019 | Immune checkpoint inhibitors in real-world treatment of older adults with non-small cell lung cancer. | Retrospective Single-centre Cohort study | Non-small cell lung cancer | IIIb, IV | Monotherapy | Nivolumab; Pembrolizumab; Atezolizumab | 37 | 75 | 49.3 | General irAE |
| Music 2019 | Serum PD-1 is elevated after pembrolizumab treatment but has no predictive value. | Prospective  Single-centre Clinical trial subset analysis | Pan-cancer | Not specified | Monotherapy | Pembrolizumab | 14 | 24 | 58.3 | General irAE |
| Music 2020 | Predicting response and toxicity to PD-1 inhibition using serum autoantibodies identified from immuno-mass spectrometry. | Prospective  Single-centre Clinical trial subset analysis | Pan-cancer | Not specified | Monotherapy | Pembrolizumab | 14 | 78 | 17.9 | General irAE |
| Nakahama 2018 | Association between imaging findings of airway obstruction adjacent to lung tumors and the onset of interstitial lung disease after nivolumab. | Retrospective  Multi-centre  Cohort study | Non-small cell lung cancer | Advanced or recurrent | Monotherapy | Nivolumab | 25 | 201 | 12.4 | Pulmonary |
| Nakamura 2019 | Correlation between blood cell count and outcome of melanoma patients treated with anti-PD-1 antibodies. | Retrospective Single-centre Cohort study | Melanoma | Unresectable M0, M1a-c | Monotherapy | Nivolumab; Pembrolizumab | 26 | 45 | 57.8 | General irAE |
| Nakanishi 2019 | Pre-existing interstitial lung abnormalities are risk factors for immune checkpoint inhibitor-induced interstitial lung disease in non-small cell lung cancer. | Retrospective Single-centre Cohort study | Non-small cell lung cancer | III, IIIb, IV, advanced or recurrent | Monotherapy | Nivolumab; Pembrolizumab | 14 | 83 | 16.9 | Pulmonary (grade specific) |
| Nakano 2020 | Correlation between cutaneous adverse events and prognosis in patients with melanoma treated with nivolumab: A single institutional retrospective study. | Retrospective Single-centre Cohort study | Melanoma | III, IV | Monotherapy | Nivolumab | 61 | 128 | 47.7 | Skin |
| Naqash 2020 | Outcomes associated with immune-related adverse events in metastatic non-small cell lung cancer treated with nivolumab: A pooled exploratory analysis from a global cohort. | Retrospective  Multi-centre  Pooled exploratory analysis | Non-small cell lung cancer | Metastatic | Monotherapy | Nivolumab | 173 | 531 | 32.6 | General irAE |
| Nishiyama 2020 | The utility of ground-glass attenuation score for anticancer treatment-related acute exacerbation of interstitial lung disease among lung cancer patients with interstitial lung disease. | Retrospective  Multi-centre  Cohort study | Non-small cell lung cancer | IIIa, IIIb, IV | Monotherapy | Nivolumab; Pembrolizumab; Atezolizumab; (non-ICI cohort included; analysis performed separately) | 7 | 48 | 14.6 | Pulmonary |

| **Author Year** | **Study Title** | **Study Design** | **Cancer Type** | **Cancer Stage (if specified)** | **ICI Type(s)** | **Specific ICI Agents** | **Patients With irAE** | **ICI Patient Population** | **Event Rate (%)** | **Type of irAE** |
| --- | --- | --- | --- | --- | --- | --- | --- | --- | --- | --- |
| Noseda 2020 | Pre-existing cardiovascular conditions as clinical predictors of myocarditis reporting with immune checkpoint inhibitors: A VigiBase study. | Retrospective  Matched case-control study | Melanoma; Non-small cell lung cancer; (Pan-cancer approach) | Not specified | Combination & monotherapy | Nivolumab; Pembrolizumab; Cemiplimab; Atezolizumab; Avelumab; Durvalumab; Ipilimumab; Combination Ipilimumab + Nivolumab; Combination Ipilimumab + Pembrolizumab | 108 | Not specified | Not assessable | Cardiac |
| Noseda 2021 | Adverse event reporting with immune checkpoint inhibitors in older patients: Age subgroup disproportionality analysis in VigiBase. | Retrospective Pharmacovigilance study | Pan-cancer | Not specified | Combination & monotherapy | Combination Anti-PD-1 + Anti-CTLA-4; Nivolumab; Pembrolizumab; Cemiplimab; Atezolizumab; Avelumab; Durvalumab; Ipilimumab | Not specified | Not specified | Not assessable | General irAE |
| Okada 2019 | Association between immune-related adverse events and clinical efficacy in patients with melanoma treated with nivolumab: A multicenter retrospective study. | Retrospective  Multi-centre  Cohort study | Melanoma | Not specified | Monotherapy | Nivolumab | 8 | 15 | 53.3 | General irAE |
| Okada 2020 (1) | Anti-thyroid antibodies and thyroid echo pattern at baseline as risk factors for thyroid dysfunction induced by anti-programmed cell death-1 antibodies: A prospective study. | Prospective  Single-centre  Cohort study | Melanoma; Non-small cell lung cancer; Renal cell carcinoma; Upper gastrointestinal cancer; Head and neck cancer; Genitourinary cancer; Hodgkin's lymphoma | Not specified | Monotherapy | Nivolumab; Pembrolizumab | 20 | 209 | 9.6 | Endocrine |
| Okada 2020 (2) | Risk factors of immune checkpoint inhibitor-related interstitial lung disease in patients with lung cancer: A single-institution retrospective study. | Retrospective Single-centre Cohort study | Lung cancer | III, IV | Monotherapy | Nivolumab; Pembrolizumab; Atezolizumab; Durvalumab | 19 | 102 | 18.6 | Pulmonary (grade specific) |
| Oren 2020 | Cardiovascular health and outcomes in cancer patients receiving immune checkpoint inhibitors. | Retrospective Single-centre Cohort study | Pan-cancer | Not specified | Monotherapy | Nivolumab; Pembrolizumab; Atezolizumab; Avelumab; Ipilimumab | 80 | 3326 | 2.4 | Cardiac |

| **Author Year** | **Study Title** | **Study Design** | **Cancer Type** | **Cancer Stage (if specified)** | **ICI Type(s)** | **Specific ICI Agents** | **Patients With irAE** | **ICI Patient Population** | **Event Rate (%)** | **Type of irAE** |
| --- | --- | --- | --- | --- | --- | --- | --- | --- | --- | --- |
| Osawa 2021 | Association between time to treatment failure and peripheral eosinophils in patients with non-small cell lung cancer treated with immune checkpoint inhibitors. | Prospective  Multi-centre  Cohort study | Non-small cell lung cancer | IIIa-c, IVa-b | Combination & monotherapy | Nivolumab; Pembrolizumab; Atezolizumab; Durvalumab; Combination Ipilimumab + Nivolumab | 35 | 259 | 13.5 | General irAE |
| Osipov 2020 | Tumor mutational burden, toxicity, and response of immune checkpoint inhibitors targeting PD(L)1, CTLA-4, and combination: A meta-regression analysis. | Systematic review, meta-analysis & meta-regression | Pan-cancer | Not specified | Combination & monotherapy | Anti-PD-1; Anti-PD-L1; Anti-CTLA-4; Combination Anti-PD1/L1 + Anti-CTLA-4; Nivolumab; Pembrolizumab; Cemiplimab; Atezolizumab; Avelumab; Durvalumab; Ipilimumab; Combination Ipilimumab + Nivolumab; Combination Ipilimumab + Pembrolizumab; Tremelimumab; Combination Tremelimumab + Durvalumab | 1712 | 9728 | 17.6 | High grade irAE |
| Osorio 2017 | Antibody-mediated thyroid dysfunction during T-cell checkpoint blockade in patients with non-small-cell lung cancer. | Prospective  Single-centre Clinical trial subset analysis | Non-small cell lung cancer | IV | Monotherapy | Pembrolizumab | 10 | 51 | 19.6 | Endocrine |
| Owen 2018 | Incidence, risk factors, and effect on survival of immune-related adverse events in patients with non-small-cell lung cancer. | Retrospective Single-centre Cohort study | Non-small cell lung cancer | Not specified | Monotherapy | Nivolumab; Pembrolizumab; Atezolizumab | 27 | 91 | 29.7 | General irAE |
|  |  |  |  |  |  |  | 9 |  | 9.9 | Pulmonary |
| Oyanagi 2019 | Predictive value of serum protein levels in patients with advanced non-small cell lung cancer treated with nivolumab. | Prospective  Single-centre  Cohort study | Non-small cell lung cancer | III, IV | Monotherapy | Nivolumab | 32 | 70 | 45.7 | General irAE |
| Oyanagi 2021 | Bloodborne cytokines for predicting clinical benefits and immune-related adverse events in advanced non-small cell lung cancer treated with anti-programmed cell death 1 inhibitors. | Prospective  Single-centre  Cohort study | Non-small cell lung cancer | III, IV | Monotherapy | Nivolumab; Pembrolizumab | 17 | 63 | 27.0 | General irAE |

| **Author Year** | **Study Title** | **Study Design** | **Cancer Type** | **Cancer Stage (if specified)** | **ICI Type(s)** | **Specific ICI Agents** | **Patients With irAE** | **ICI Patient Population** | **Event Rate (%)** | **Type of irAE** |
| --- | --- | --- | --- | --- | --- | --- | --- | --- | --- | --- |
| Park 2020 | Immune-related adverse events on body CT in patients with small-cell lung cancer treated with immune-checkpoint inhibitors. | Retrospective Single-centre Cohort study | Small cell lung cancer | Limited, extensive | Combination & monotherapy | Nivolumab; Pembrolizumab; Atezolizumab; Combination Ipilimumab + Nivolumab | 19 | 53 | 35.8 | Pulmonary |
| Pavan 2019 | Peripheral blood markers identify risk of immune-related toxicity in advanced non-small cell lung cancer treated with immune-checkpoint inhibitors. | Retrospective  Multi-centre  Cohort study | Non-small cell lung cancer | Advanced | Monotherapy | Nivolumab; Pembrolizumab; Atezolizumab | 60 | 184 | 32.6 | General irAE |
| Peng 2020 | Peripheral blood markers predictive of outcome and immune-related adverse events in advanced non-small cell lung cancer treated with PD-1 inhibitors. | Retrospective Single-centre Cohort study | Non-small cell lung cancer | IIIb, IV | Monotherapy | Nivolumab; Pembrolizumab; Sintilimab; Toripalimab | 39 | 102 | 38.2 | General irAE |
| Pico de Coaña 2017 | Ipilimumab treatment decreases monocytic MDSCs and increases CD8 effector memory T cells in long-term survivors with advanced melanoma. | Prospective  Multi-centre  Cohort study | Pan-cancer | IV, M1a-c | Monotherapy | Ipilimumab | 23 | 43 | 53.5 | General irAE |
| Pistillo 2019 | Soluble CTLA-4 as a favorable predictive biomarker in metastatic melanoma patients treated with ipilimumab: An Italian melanoma intergroup study. | Retrospective  Multi-centre  Cohort study | Melanoma | IV | Monotherapy | Ipilimumab | 28 | 113 | 24.8 | General irAE |
| Pollack 2019 | Baseline TSH level is associated with risk of anti-PD-1-induced thyroid dysfunction. | Retrospective Single-centre Cohort study | Melanoma | IV | Combination & monotherapy | Nivolumab; Pembrolizumab; Combination Ipilimumab + Nivolumab; Combination Ipilimumab + Pembrolizumab | 22 | 73 | 30.1 | Endocrine |
| Pollack 2020 | Immune checkpoint inhibitor-induced thyroid dysfunction is associated with higher body mass index. | Retrospective Single-centre Cohort study | Lung cancer; Gastrointestinal cancer; Genitourinary cancer | III, IV | Monotherapy | Nivolumab; Pembrolizumab; Atezolizumab; Durvalumab | 72 | 185 | 38.9 | Endocrine |
| Popinat 2019 | Sub-cutaneous fat mass measured on multislice computed tomography of pretreatment PET/CT is a prognostic factor of stage IV non-small cell lung cancer treated by nivolumab. | Retrospective Single-centre Cohort study | Non-small cell lung cancer | IV | Monotherapy | Nivolumab | 21 | 234 | 9.0 | High grade irAE |
| Presotto 2020 | Endocrine toxicity in cancer patients treated with nivolumab or pembrolizumab: Results of a large multicentre study. | Retrospective  Multi-centre  Cohort study | Pan-cancer | Not specified | Monotherapy | Nivolumab; Pembrolizumab | 54 | 179 | 30.2 | Endocrine |

| **Author Year** | **Study Title** | **Study Design** | **Cancer Type** | **Cancer Stage (if specified)** | **ICI Type(s)** | **Specific ICI Agents** | **Patients With irAE** | **ICI Patient Population** | **Event Rate (%)** | **Type of irAE** |
| --- | --- | --- | --- | --- | --- | --- | --- | --- | --- | --- |
| Purde 2021 | Presence of autoantibodies in serum does not impact the occurrence of immune checkpoint inhibitor-induced hepatitis in a prospective cohort of cancer patients. | Prospective  Single-centre Observational cohort study | Melanoma; Non-small cell lung cancer | Advanced or metastatic | Combination & monotherapy | Anti-PD-1; Anti-PD-L1; Anti-CTLA-4; Combination Anti-PD1/L1 + Anti-CTLA-4 | 11 | 131 | 8.4 | Gastrointestinal |
| Ramos-Levi 2019 | Nivolumab-induced thyroid dysfunction in patients with lung cancer. | Retrospective Single-centre Cohort study | Non-small cell lung cancer | IIIb, IV | Monotherapy | Nivolumab | 9 | 40 | 22.5 | Endocrine |
| Refae 2020 | Germinal immunogenetics predict treatment outcome for PD-1/PD-L1 checkpoint inhibitors. | Prospective  Single-centre Clinical trial subset analysis | Pan-cancer | Advanced | Monotherapy | Nivolumab; Pembrolizumab; Atezolizumab; Avelumab; Durvalumab | 79 | 94 | 84.0 | General irAE |
| Reuss 2021 | Pretreatment lung function and checkpoint inhibitor pneumonitis in NSCLC. | Retrospective Single-centre Cohort study | Non-small cell lung cancer | I to IV | Combination & monotherapy | Nivolumab; Pembrolizumab; Durvalumab; Combination Ipilimumab + Nivolumab | 9 | 43 | 20.9 | Pulmonary |
| Richter 2018 | Brief report: Cancer immunotherapy in patients with preexisting rheumatic disease: The Mayo Clinic Experience. | Retrospective Single-centre Cohort study | Pan-cancer | Mostly IV | Combination & monotherapy | Nivolumab; Pembrolizumab; Ipilimumab; Combination Ipilimumab + Nivolumab; Combination Ipilimumab + Pembrolizumab | 6 | 16 | 37.5 | General irAE |
| Richtig 2018 | Body mass index may predict the response to ipilimumab in metastatic melanoma: An observational multi-centre study. | Retrospective  Multi-centre  Cohort study | Melanoma | Mostly M1c | Monotherapy | Ipilimumab | 38 | 76 | 50.0 | General irAE |
| Rogado 2020 | Effect of excess weight and immune-related adverse events on the efficacy of cancer immunotherapy with anti-PD-1 antibodies. | Retrospective Single-centre Observational cohort study | Pan-cancer | Advanced | Monotherapy | Nivolumab; Pembrolizumab | 44 | 132 | 33.3 | General irAE |
| Romanski 2020 | Characterization of risk factors and efficacy of medical management of immune-related hepatotoxicity in real-world patients with metastatic melanoma treated with immune checkpoint inhibitors. | Retrospective Single-centre Cohort study | Melanoma | M1a-c | Combination & monotherapy | Nivolumab; Pembrolizumab; Ipilimumab; Combination Ipilimumab + Nivolumab | 43 | 521 | 8.3 | Gastrointestinal (grade specific) |
| Rubino 2021 | Endocrine-related adverse events in a large series of cancer patients treated with anti-PD1 therapy. | Retrospective  Multi-centre  Cohort study | Pan-cancer | Advanced | Monotherapy | Nivolumab; Pembrolizumab | 70 | 251 | 27.9 | Endocrine |
| Sakakida 2020 | Safety and efficacy of PD-1/PD-L1 blockade in patients with preexisting antinuclear antibodies. | Retrospective Single-centre Cohort study | Pan-cancer | Metastatic or unresectable | Monotherapy | Nivolumab; Pembrolizumab; Atezolizumab; Durvalumab | 73 | 191 | 38.2 | General irAE |

| **Author Year** | **Study Title** | **Study Design** | **Cancer Type** | **Cancer Stage (if specified)** | **ICI Type(s)** | **Specific ICI Agents** | **Patients With irAE** | **ICI Patient Population** | **Event Rate (%)** | **Type of irAE** |
| --- | --- | --- | --- | --- | --- | --- | --- | --- | --- | --- |
| Sakata 2019 | The association between tumor burden and severe immune-related adverse events in non-small cell lung cancer patients responding to immune-checkpoint inhibitor treatment. | Retrospective Single-centre Cohort study | Non-small cell lung cancer | II, III, IV, recurrent | Monotherapy | Nivolumab; Pembrolizumab; Atezolizumab | 15 | 42 | 35.7 | High grade irAE |
| Salem 2018 | Cardiovascular toxicities associated with immune checkpoint inhibitors: An observational, retrospective, pharmacovigilance study. | Retrospective Pharmacovigilance study | Pan-cancer | Not specified | Combination & monotherapy | Combination Anti-PD1/L1 + Anti-CTLA-4; Nivolumab; Pembrolizumab; Atezolizumab; Avelumab; Durvalumab; Ipilimumab; Tremelimumab | Not specified | Not specified | Not assessable | Cardiac |
| Samani 2020 | Impact of age on the toxicity of immune checkpoint inhibition. | Retrospective  Multi-centre  Cohort study | Melanoma; Non-small cell lung cancer; Renal cell carcinoma; (Pan-cancer approach) | Advanced or metastatic | Combination & monotherapy | Nivolumab; Pembrolizumab; Ipilimumab; Combination Ipilimumab + Nivolumab | 270 | 448 | 60.3 | General irAE |
|  |  |  |  |  |  |  | 72 |  | 16.1 | High grade irAE |
| Sanghavi 2021 | Nivolumab exposure-response analysis for adjuvant treatment of melanoma supporting a change in posology. | Retrospective Clinical trial subset analysis | Melanoma | Adjuvant M0, M1a-c | Monotherapy | Nivolumab | 146 | 448 | 32.6 | High grade irAE |
| Sasson 2020 | Mucosal-associated invariant T (MAIT) cells are activated in the gastrointestinal tissue of patients with combination ipilimumab and nivolumab therapy-related colitis in a pathology distinct from ulcerative colitis. | Prospective  Multi-centre Experimental study | Melanoma | M1a-d | Combination only | Combination Ipilimumab + Nivolumab | 29 | 58 | 50.0 | Gastrointestinal |
| Sato 2018 | Correlation between immune-related adverse events and efficacy in non-small cell lung cancer treated with nivolumab. | Retrospective Single-centre Cohort study | Non-small cell lung cancer | IIIb, IV, postoperative relapse | Monotherapy | Nivolumab | 11 | 38 | 28.9 | General irAE |
| Sawada 2020 | Non-alcoholic fatty liver disease is a potential risk factor for liver injury caused by immune checkpoint inhibitor. | Retrospective Single-centre Cohort study | Pan-cancer | Advanced | Monotherapy | Nivolumab; Pembrolizumab | 8 | 135 | 5.9 | Gastrointestinal (grade specific) |
| Sbardella 2020 | Thyroid disorders in programmed death 1 inhibitor-treated patients: Is previous therapy with tyrosine kinase inhibitors a predisposing factor?. | Retrospective Single-centre Cohort study | Melanoma; Non-small cell lung cancer; Renal cell carcinoma (Pan cancer approach) | Advanced | Monotherapy | Nivolumab; Pembrolizumab | 29 | 126 | 23.0 | Endocrine |
| Schilling 2021 | Development of a flow cytometry assay to predict immune checkpoint blockade-related complications. | Prospective  Single-centre Experimental study | Melanoma | II, III, IV | Monotherapy | Nivolumab; Ipilimumab | 16 | 83 | 19.3 | Gastrointestinal |

| **Author Year** | **Study Title** | **Study Design** | **Cancer Type** | **Cancer Stage (if specified)** | **ICI Type(s)** | **Specific ICI Agents** | **Patients With irAE** | **ICI Patient Population** | **Event Rate (%)** | **Type of irAE** |
| --- | --- | --- | --- | --- | --- | --- | --- | --- | --- | --- |
| Seethapathy 2019 | The incidence, causes, and risk factors of acute kidney injury in patients receiving immune checkpoint inhibitors. | Retrospective Single-centre Cohort study | Pan-cancer | Advanced | Combination & monotherapy | Nivolumab; Pembrolizumab; Atezolizumab; Avelumab; Durvalumab; Ipilimumab; Combination Ipilimumab + Nivolumab | 82 | 1016 | 8.1 | Renal |
| Seethapathy 2020 | Incidence and clinical features of immune-related acute kidney injury in patients receiving programmed cell death ligand-1 inhibitors. | Retrospective Single-centre Cohort study | Pan-cancer | Not specified | Monotherapy | Atezolizumab; Avelumab; Durvalumab | 104 | 599 | 17.4 | Renal |
| Serna-Higuita 2021 | Association between immune-related adverse events and survival in 319 stage IV melanoma patients treated with PD-1-based immunotherapy: An approach based on clinical chemistry. | Retrospective Single-centre Cohort study | Melanoma | IV | Combination & monotherapy | Nivolumab; Pembrolizumab; Combination Ipilimumab + Nivolumab | 169 | 319 | 53.0 | General irAE |
| Shah 2020 | Demographic factors associated with toxicity in patients treated with anti-programmed cell death-1 therapy. | Retrospective Single-centre Cohort study | Pan-cancer | Adjuvant or metastatic disease | Combination & monotherapy | Anti-PD-1; Combination Ipilimumab + Nivolumab | 226 | 455 | 49.7 | General irAE |
| Shaverdian 2017 | Previous radiotherapy and the clinical activity and toxicity of pembrolizumab in the treatment of non-small-cell lung cancer: A secondary analysis of the KEYNOTE-001 phase 1 trial. | Retrospective Single-centre Clinical trial subset analysis | Non-small cell lung cancer | I to IV | Monotherapy | Pembrolizumab | 44 | 97 | 45.4 | Pulmonary (grade specific) |
| Shemesh 2020 | Pan-cancer population pharmacokinetics and exposure-safety and -efficacy analyses of atezolizumab in patients with high tumor mutational burden. | Retrospective  Multi-centre  Clinical trial subset analysis | Pan-cancer | Mostly locally advanced or metastatic | Monotherapy | Atezolizumab | 167 | 986 | 16.9 | High grade irAE |
| Shi 2021 | Correlations between peripheral blood biomarkers and clinical outcomes in advanced non-small cell lung cancer patients who received immunotherapy-based treatments. | Retrospective Single-centre Cohort study | Non-small cell lung cancer | III, IV | Monotherapy | Anti-PD-L1; (non-ICI cohort included; analysis performed separately) | 14 | 32 | 43.8 | General irAE |
| Shibaki 2020 | Association of immune-related pneumonitis with the presence of preexisting interstitial lung disease in patients with non-small lung cancer receiving anti-programmed cell death 1 antibody. | Retrospective Single-centre Cohort study | Non-small cell lung cancer | III, IV, recurrent | Monotherapy | Nivolumab; Pembrolizumab | 36 | 331 | 10.9 | Pulmonary |
| Shimizu 2020 | Clinical impact of sarcopenia and inflammatory/nutritional markers in patients with unresectable metastatic urothelial carcinoma treated with pembrolizumab. | Retrospective Single-centre Cohort study | Urothelial carcinoma | Metastatic or unresectable | Monotherapy | Pembrolizumab | 10 | 27 | 37.0 | General irAE |
|  |  |  |  |  |  |  | 5 |  | 18.5 | High grade irAE |

| **Author Year** | **Study Title** | **Study Design** | **Cancer Type** | **Cancer Stage (if specified)** | **ICI Type(s)** | **Specific ICI Agents** | **Patients With irAE** | **ICI Patient Population** | **Event Rate (%)** | **Type of irAE** |
| --- | --- | --- | --- | --- | --- | --- | --- | --- | --- | --- |
| Shimozaki 2020 | Multiple immune-related adverse events and anti-tumor efficacy: Real-world data on various solid tumors. | Retrospective Single-centre Cohort study | Melanoma; Non-small cell lung cancer; Renal cell carcinoma; Gastric cancer | Recurrent or metastatic | Monotherapy | Nivolumab; Pembrolizumab; Atezolizumab | 108 | 212 | 50.9 | General irAE |
| Shimozaki 2021 | Analysis of risk factors for immune-related adverse events in various solid tumors using real-world data. | Retrospective Single-centre Cohort study | Melanoma; Non-small cell lung cancer; Renal cell carcinoma; Gastric cancer | Metastatic | Monotherapy | Nivolumab; Pembrolizumab; Atezolizumab | 118 | 247 | 47.8 | General irAE |
| Sibille 2021 | White blood cells in patients treated with programmed cell death-1 inhibitors for non-small cell lung cancer. | Retrospective Single-centre Cohort study | Non-small cell lung cancer | II, III, IV | Monotherapy | Nivolumab; Pembrolizumab; Atezolizumab; Durvalumab | 46 | 191 | 24.1 | General irAE |
| Siddiqui 2021 | Predicting development of ipilimumab-induced hypophysitis: Utility of T4 and TSH index but not TSH. | Retrospective Single-centre Cohort study | Melanoma | Advanced | Combination & monotherapy | Ipilimumab; Combination Ipilimumab + Nivolumab | 25 | 308 | 8.1 | Endocrine |
| Smith 2020 | Combined regimen immune checkpoint inhibitor-associated hepatitis: Experience from a North American multicenter cohort. | Retrospective Single-centre Cohort study | Melanoma | III, IV | Combination only | Combination Ipilimumab + Nivolumab | 32 | 63 | 50.8 | Gastrointestinal |
| Snyders 2019 | Ipilimumab-induced hypophysitis, a single academic center experience. | Retrospective Single-centre Cohort study | Melanoma | Metastatic or unresectable | Monotherapy | Ipilimumab | 15 | 117 | 12.8 | Endocrine |
| Sonehara 2021 | The role of immune-related adverse events in prognosis and efficacy prediction for patients with non-small cell lung cancer treated with immunotherapy: A retrospective clinical analysis. | Retrospective Single-centre Cohort study | Non-small cell lung cancer | Not specified | Monotherapy | Nivolumab; Pembrolizumab; Atezolizumab | 25 | 80 | 31.3 | General irAE |
| Sorah 2021 | Incidence and prediction of immune checkpoint inhibitor-related nephrotoxicity. | Retrospective Single-centre Cohort study | Pan-cancer | All stages | Combination & monotherapy | Nivolumab; Pembrolizumab; Atezolizumab; Ipilimumab; Combination Ipilimumab + Nivolumab | 123 | 1766 | 7.0 | Renal |
| Soularue 2018 | Enterocolitis due to immune checkpoint inhibitors: A systematic review. | Systematic review | Pan-cancer | Not specified | Monotherapy | Anti-PD-1; Anti-CTLA-4; Nivolumab; Pembrolizumab; Atezolizumab; Avelumab; Durvalumab; Ipilimumab; Tremelimumab; Other ICI (not specified) | Not specified | Not specified | Not assessable | Gastrointestinal |

| **Author Year** | **Study Title** | **Study Design** | **Cancer Type** | **Cancer Stage (if specified)** | **ICI Type(s)** | **Specific ICI Agents** | **Patients With irAE** | **ICI Patient Population** | **Event Rate (%)** | **Type of irAE** |
| --- | --- | --- | --- | --- | --- | --- | --- | --- | --- | --- |
| Stein 2021 | Acute kidney injury in patients treated with anti-programmed death receptor-1 for advanced melanoma: A real-life study in a single-centre cohort. | Retrospective Single-centre Cohort study | Melanoma | IIIb-c, IV | Monotherapy | Nivolumab; Pembrolizumab | 41 | 239 | 17.2 | Renal |
| Suazo-Zepeda 2021 | Risk factors for adverse events induced by immune checkpoint inhibitors in patients with non-small-cell lung cancer: A systematic review and meta-analysis. | Systematic review & meta-analysis | Non-small cell lung cancer | All stages | Monotherapy | Nivolumab; Pembrolizumab; Cemiplimab; Atezolizumab; Avelumab; Durvalumab | 1653 | 6696 | 24.7 | Pulmonary (including high grade) |
| Sugano 2020 | Immune checkpoint inhibitor-associated interstitial lung diseases correlate with better prognosis in patients with advanced non-small-cell lung cancer. | Retrospective Single-centre Cohort study | Non-small cell lung cancer | Advanced | Monotherapy | Nivolumab; Pembrolizumab; Atezolizumab | 39 | 130 | 30.0 | Pulmonary |
| Sugisaka 2020 | Relationship between programmed cell death protein ligand 1 expression and immune-related adverse events in non-small-cell lung cancer patients treated with pembrolizumab. | Retrospective Single-centre Cohort study | Non-small cell lung cancer | IIIb, IV | Monotherapy | Pembrolizumab | 31 | 44 | 70.5 | General irAE |
| Sukari 2019 | Cancer site and adverse events induced by immune checkpoint inhibitors: A retrospective analysis of real-life experience at a single institution. | Retrospective Single-centre Cohort study | Pan-cancer | Not specified | Monotherapy | Nivolumab; Pembrolizumab | 101 | 168 | 60.1 | General irAE |
| Suzuki 2017 | Nivolumab-related myasthenia gravis with myositis and myocarditis in Japan. | Retrospective  Multi-centre  Cohort study | Pan-cancer | Not specified | Monotherapy | Nivolumab; Ipilimumab | 12 | 10277 | 0.1 | Neurological |
| Suzuki 2020 | Assessment of immune-related interstitial lung disease in patients with NSCLC treated with immune checkpoint inhibitors: A multicenter prospective study. | Prospective  Multi-centre  Cohort study | Non-small cell lung cancer | Unresectable stage IIIa, IIIb, IV | Monotherapy | Nivolumab; Pembrolizumab | 20 | 138 | 14.5 | Pulmonary |
| Tahir 2019 | Autoimmune antibodies correlate with immune checkpoint therapy-induced toxicities. | Prospective  Single-centre Experimental study | Pan-cancer | Not specified | Monotherapy | Ipilimumab | 8 | 29 | 27.6 | Endocrine |
|  |  |  |  |  |  |  | 12 | 40 | 30.0 | Pulmonary |
| Takada 2020 | Predictors of the onset of type 1 diabetes obtained from real-world data analysis in cancer patients treated with immune checkpoint inhibitors. | Retrospective  Multi-centre  Cohort study | Pan-cancer | Not specified | Monotherapy | Nivolumab; Pembrolizumab; Atezolizumab; Durvalumab | 303 | 10562 | 2.9 | Endocrine |
| Takahashi 2020 | Intracellular accumulation of PD-1 molecules in circulating T lymphocytes in advanced malignant melanoma: An implication for immune evasion mechanism. | Prospective  Single-centre Experimental study | Melanoma | Advanced | Monotherapy | Nivolumab | 1 | 6 | 16.7 | Skin |

| **Author Year** | **Study Title** | **Study Design** | **Cancer Type** | **Cancer Stage (if specified)** | **ICI Type(s)** | **Specific ICI Agents** | **Patients With irAE** | **ICI Patient Population** | **Event Rate (%)** | **Type of irAE** |
| --- | --- | --- | --- | --- | --- | --- | --- | --- | --- | --- |
| Takenaka 2021 | Predictive impact of sarcopenia in solid cancers treated with immune checkpoint inhibitors: A meta-analysis. | Systematic review & meta-analysis | Pan-cancer | Not specified | Monotherapy | Nivolumab; Pembrolizumab; Cemiplimab; Atezolizumab; Avelumab; Durvalumab; Ipilimumab | Not specified | 2501 | Not assessable | High grade irAE |
| Tamiya 2017 | Correlation of radiation pneumonitis history before nivolumab with onset of interstitial lung disease and progression-free survival of patients with pre-treated advanced non-small cell lung cancer. | Retrospective  Multi-centre  Cohort study | Non-small cell lung cancer | Advanced | Monotherapy | Nivolumab | 34 | 201 | 16.9 | Pulmonary |
| Teraoka 2017 | Early immune-related adverse events and association with outcome in advanced non-small cell lung cancer patients treated with nivolumab: A prospective cohort study. | Prospective  Single-centre  Cohort study | Non-small cell lung cancer | IIIb, IV | Monotherapy | Nivolumab | 19 | 43 | 44.2 | General irAE |
| Tison 2019 | Safety and efficacy of immune checkpoint inhibitors in patients with cancer and preexisting autoimmune disease: A nationwide, multicenter cohort study. | Retrospective  Multi-centre  Cohort study | Pan-cancer | Not specified | Combination & monotherapy | Nivolumab; Pembrolizumab; Atezolizumab; Avelumab; Ipilimumab; Combination Ipilimumab + Nivolumab | 47 | 112 | 42.0 | General irAE |
| Toi 2018 | Association of immune-related adverse events with clinical benefit in patients with advanced non-small-cell lung cancer treated with nivolumab. | Retrospective Single-centre Cohort study | Non-small cell lung cancer | Advanced | Monotherapy | Nivolumab | 28 | 70 | 40.0 | General irAE |
| Toi 2019 | Profiling preexisting antibodies in patients treated with anti-PD-1 therapy for advanced non-small cell lung cancer. | Retrospective Single-centre Cohort study | Non-small cell lung cancer | Advanced | Monotherapy | Nivolumab; Pembrolizumab | 66 | 137 | 48.2 | General irAE |
|  |  |  |  |  |  |  | 9 |  | 6.6 | High grade irAE |
| Tone 2019 | High mortality and poor treatment efficacy of immune checkpoint inhibitors in patients with severe grade checkpoint inhibitor pneumonitis in non-small cell lung cancer. | Retrospective Single-centre Cohort study | Non-small cell lung cancer | IV, unresectable stage III, or postoperative recurrent | Monotherapy | Nivolumab; Pembrolizumab; Atezolizumab | 22 | 71 | 31.0 | Pulmonary (grade specific) |
| Tsukagoshi 2020 | Skeletal muscle mass predicts the outcome of nivolumab treatment for non-small cell lung cancer. | Retrospective Single-centre Cohort study | Non-small cell lung cancer | IIIb, IV or recurrent | Monotherapy | Nivolumab | 5 | 30 | 16.7 | General irAE |
| Ueda 2019 | The impact of antibiotics on prognosis of metastatic renal cell carcinoma in japanese patients treated with immune checkpoint inhibitors. | Retrospective Single-centre Cohort study | Renal cell carcinoma | T1-4 | Combination & monotherapy | Nivolumab; Combination Ipilimumab + Nivolumab | 11 | 31 | 35.5 | General irAE |

| **Author Year** | **Study Title** | **Study Design** | **Cancer Type** | **Cancer Stage (if specified)** | **ICI Type(s)** | **Specific ICI Agents** | **Patients With irAE** | **ICI Patient Population** | **Event Rate (%)** | **Type of irAE** |
| --- | --- | --- | --- | --- | --- | --- | --- | --- | --- | --- |
| Usyk 2021 | Bacteroides vulgatus and Bacteroides dorei predict immune-related adverse events in immune checkpoint blockade treatment of metastatic melanoma. | Prospective  Single-centre  Cohort study | Melanoma | III, IV | Combination & monotherapy | Nivolumab; Combination Ipilimumab + Nivolumab | Not specified | 27 | Not assessable | High grade irAE |
| Valpione 2018 | Sex and interleukin-6 are prognostic factors for autoimmune toxicity following treatment with anti-CTLA4 blockade. | Prospective  Single-centre Observational study | Melanoma | Metastatic | Monotherapy | Ipilimumab | 65 | 140 | 46.4 | General irAE |
| Verheijden 2020 | Lower risk of severe checkpoint inhibitor toxicity in more advanced disease. | Retrospective  Multi-centre  Cohort study | Melanoma | III, IV M1a-d | Monotherapy | Nivolumab; Pembrolizumab | 111 | 819 | 13.6 | General irAE |
| Wang 2017 | Incidence of immune checkpoint inhibitor-related colitis in solid tumor patients: A systematic review and meta-analysis. | Systematic review & meta-analysis | Pan-cancer | Advanced | Combination & monotherapy | Nivolumab; Pembrolizumab; Atezolizumab; Ipilimumab; Combination Ipilimumab + Nivolumab | 280 | 8863 | 3.2 | Gastrointestinal (grade specific) |
| Wang 2018 | Immune-checkpoint inhibitor-induced diarrhea and colitis in patients with advanced malignancies: Retrospective review at MD Anderson. | Retrospective Single-centre Cohort study | Pan-cancer | III, IV | Combination & monotherapy | Nivolumab; Pembrolizumab; Atezolizumab; Ipilimumab; Combination Ipilimumab + Nivolumab | 117 | 327 | 35.8 | Gastrointestinal |
| Wang 2020 | Sarcopenia affects clinical efficacy of immune checkpoint inhibitors in non-small cell lung cancer patients: A systematic review and meta-analysis. | Systematic review & meta-analysis | Non-small cell lung cancer | Advanced, recurrent or metastatic | Monotherapy | Nivolumab; Pembrolizumab; Atezolizumab | Not specified | 576 | Not assessable | General irAE |
| Wang 2021 | Plasma cytokines interleukin-18 and C-X-C motif chemokine ligand 10 are indicative of the anti-programmed cell death protein-1 treatment response in lung cancer patients. | Prospective  Single-centre  Cohort study | Lung cancer | III, IV | Monotherapy | Nivolumab; Pembrolizumab; Atezolizumab | 17 | 32 | 53.1 | General irAE |
| Watanabe 2020 | Prognostic significance of the radiologic features of pneumonitis induced by anti-PD-1 therapy. | Retrospective  Multi-centre  Cohort study | Non-small cell lung cancer | Advanced | Monotherapy | Nivolumab; Pembrolizumab | 33 | 231 | 14.3 | Pulmonary |
| Welaya 2020 | Geriatric assessment and treatment outcomes in older adults with cancer receiving immune checkpoint inhibitors. | Retrospective Single-centre Cohort study | Pan-cancer | III, IV | Monotherapy | Nivolumab; Pembrolizumab; Atezolizumab; Ipilimumab | 11 | 28 | 39.3 | General irAE |
| Wongvibulsin 2021 | Epidemiology and risk factors for the development of cutaneous toxicities in patients treated with immune-checkpoint inhibitors: A United States population-level analysis. | Retrospective Population level matched case-control study | Pan-cancer | Not specified | Combination & monotherapy | Anti-PD-1; Anti-PD-L1; Anti-CTLA-4; Combination Anti-PD1/L1 + Anti-CTLA-4 | 2171 | 8637 | 25.1 | Skin (grade specific) |

| **Author Year** | **Study Title** | **Study Design** | **Cancer Type** | **Cancer Stage (if specified)** | **ICI Type(s)** | **Specific ICI Agents** | **Patients With irAE** | **ICI Patient Population** | **Event Rate (%)** | **Type of irAE** |
| --- | --- | --- | --- | --- | --- | --- | --- | --- | --- | --- |
| Wu 2017 | PD-1 inhibitors increase the incidence and risk of pneumonitis in cancer patients in a dose-independent manner: A meta-analysis. | Systematic review & meta-analysis of phase II/III clinical trials | Pan-cancer | Not specified | Combination & monotherapy | Anti-PD-1; Combination Anti-PD-1 + Anti-CTLA-4; Nivolumab; Pembrolizumab | Not specified | 6360 | Not assessable | General irAE |
| Yamaguchi 2018 | Pre-existing pulmonary fibrosis is a risk factor for anti-PD-1-related pneumonitis in patients with non-small cell lung cancer: A retrospective analysis. | Retrospective Single-centre Cohort study | Non-small cell lung cancer | Mostly IV | Monotherapy | Nivolumab; Pembrolizumab | 18 | 123 | 14.6 | Pulmonary |
| Yamaguchi 2021 (1) | Preexisting autoimmune disease is a risk factor for immune-related adverse events: A meta-analysis. | Meta-analysis | Non-small cell lung cancer; Head and neck cancer; Gastric cancer | Not specified | Monotherapy | Nivolumab | 22 | 188 | 11.7 | Pulmonary |
| Yamaguchi 2021 (2) | Pre-existing interstitial lung disease is associated with onset of nivolumab-induced pneumonitis in patients with solid tumors: A retrospective analysis. | Retrospective Single-centre Cohort study | Pan-cancer | III, IV, advanced or metastatic | Combination & monotherapy | Nivolumab; Pembrolizumab; Atezolizumab; Combination Ipilimumab + Anti-PD-1 | 1725 | 3284 | 52.5 | General irAE |
| Yamamoto 2021 (1) | Clinical features of immune-mediated hepatotoxicity induced by immune checkpoint inhibitors in patients with cancers. | Retrospective Single-centre Cohort study | Pan-cancer | Advanced | Combination & monotherapy | Nivolumab; Pembrolizumab; Atezolizumab; Durvalumab; Ipilimumab; Combination Ipilimumab + Nivolumab | 21 | 250 | 8.4 | Gastrointestinal (grade specific) |
| Yamamoto 2021 (2) | Real-world safety of nivolumab in patients with non-small-cell lung cancer in Japan: Postmarketing surveillance. | Retrospective  Multi-centre  Post-marketing surveillance study | Non-small cell lung cancer | Advanced or recurrent | Monotherapy | Nivolumab | 1697 | 3601 | 47.1 | General irAE |
| Yamauchi 2019 | Incidence, features, and prognosis of immune-related adverse events involving the thyroid gland induced by nivolumab. | Retrospective Single-centre Cohort study | Pan-cancer | Not specified | Monotherapy | Nivolumab | 67 | 200 | 33.5 | Endocrine |
| Yano 2018 | Nivolumab-induced thyroid dysfunction lacking antithyroid antibody is frequently evoked in Japanese patients with malignant melanoma. | Retrospective Single-centre Cohort study | Melanoma | II, III, IV | Monotherapy | Nivolumab | 7 | 24 | 29.2 | Endocrine |
| Ye 2021 | Checkpoint-blocker-induced autoimmunity is associated with favourable outcome in metastatic melanoma and distinct T-cell expression profiles. | Prospective  Multi-centre Experimental study | Melanoma | Metastatic | Combination & monotherapy | Nivolumab; Pembrolizumab; Combination Ipilimumab + Nivolumab | 93 | 144 | 64.6 | General irAE |
| Yoon 2021 | Characteristics of immune-related thyroid adverse events in patients treated with PD-1/PD-L1 inhibitors. | Retrospective Single-centre Cohort study | Pan-cancer | Not specified | Monotherapy | Nivolumab; Pembrolizumab; Atezolizumab; Durvalumab | 164 | 325 | 50.5 | Endocrine |

| **Author Year** | **Study Title** | **Study Design** | **Cancer Type** | **Cancer Stage (if specified)** | **ICI Type(s)** | **Specific ICI Agents** | **Patients With irAE** | **ICI Patient Population** | **Event Rate (%)** | **Type of irAE** |
| --- | --- | --- | --- | --- | --- | --- | --- | --- | --- | --- |
| Young 2020 | Impact of body composition on outcomes from anti-PD1 +/- anti-CTLA-4 treatment in melanoma. | Prospective  Single-centre  Cohort study | Melanoma | M1a-d | Combination & monotherapy | Nivolumab; Pembrolizumab; Atezolizumab; Combination Ipilimumab + Nivolumab | Not specified | 287 | Not assessable | General irAE |
| Zeng 2020 | Effect of comorbidity on outcomes of patients with advanced non-small cell lung cancer undergoing anti-PD1 immunotherapy. | Retrospective Single-centre Cohort study | Non-small cell lung cancer | III, IV | Monotherapy | Nivolumab; Pembrolizumab; Toripalimab | 20 | 66 | 30.3 | General irAE |
| Zhai 2019 | Endocrine toxicity of immune checkpoint inhibitors: A real-world study leveraging US Food and Drug Administration adverse events reporting system. | Retrospective Pharmacovigilance study | Pan-cancer | Not specified | Combination & monotherapy | Nivolumab; Pembrolizumab; Cemiplimab; Atezolizumab; Avelumab; Durvalumab; Ipilimumab; Combination Ipilimumab + Nivolumab; Combination Ipilimumab + Pembrolizumab; Tremelimumab; Combination Tremelimumab + Durvalumab; Combination Nivolumab + Pembrolizumab + Ipilimumab | 6260 | Not specified | Not assessable | Endocrine |
| Zhang 2021 (1) | Analysis of the gut microbiota: An emerging source of biomarkers for immune checkpoint blockade therapy in non-small cell lung cancer. | Retrospective Single-centre Cohort study | Non-small cell lung cancer | Metastatic (brain) | Monotherapy | Pembrolizumab | 26 | 69 | 37.7 | General irAE |
|  |  |  |  |  |  |  | 2 |  | 2.9 | High grade irAE |
| Zhang 2021 (2) | Improved survival and disease control following pembrolizumab-induced immune-related adverse events in high PD-L1 expressing non-small cell lung cancer with brain metastases. | Prospective  Single-centre  Cohort study | Non-small cell lung cancer | III, IV | Monotherapy | Nivolumab; Pembrolizumab; Atezolizumab | 24 | 63 | 38.1 | General irAE |
| Zhao 2020 | Association between immune-related adverse events and efficacy of PD-1 inhibitors in Chinese patients with advanced melanoma. | Retrospective Single-centre Cohort study | Melanoma | M1a-d | Monotherapy | Nivolumab; Pembrolizumab | 54 | 93 | 58.1 | General irAE |

AKI, acute kidney injury; AD, autoimmune disease; CNS, central nervous system; CTLA-4, cytotoxic T-lymphocyte associated protein 4; IBD, inflammatory bowel disease; ICI, immune checkpoint inhibitor; irAE, immune-related adverse event; NSCLC, non-small cell lung cancer, PD-1, programmed death 1; programmed death ligand-1, PD-L1.

^#^Non-ICI cohort included and analysis for ICI treatment performed separately to other cancer treatments in study.
