# Supplementary material for: Cancer and treatment specific incidence rates of immune-related adverse events induced by immune checkpoint inhibitors: a systematic review
Source: Br J Cancer. 2024 Nov 3;132(1):51–7. doi: 10.1038/s41416-024-02887-1 (PMC11723908; doi:10.1038/s41416-024-02887-1)
Supplement: Supplementary file 3 — Table S3 Specific adverse events within each irAE type [file 41416_2024_2887_MOESM3_ESM.docx]

**Supplementary Table S3:** Specific adverse events/symptoms reported within each irAE type.

irAE, immune-related adverse event.

| **Type of irAE** | **Specific adverse event or symptom** |
| --- | --- |
| Cardiac irAE | Myocarditis, acute vascular events, pericarditis, pericardial disease pericardial effusion, atrial fibrillation, arrhythmias, cardiac/heart failure, vasculitis, dyspnoea |
| Endocrine irAE | Thyroid: hypothyroidism, hyperthyroidism, thyrotoxicosis, thyroiditis |
|  | Adrenal: adrenal insufficiency, isolated adrenocorticotropic hormone deficiency |
|  | Pituitary: hypophysitis |
|  | Pancreatic: type 1 diabetes mellitus, new onset and worsening type 2 diabetes |
| Gastrointestinal irAE | Colitis, diarrhoea, hepatitis, cholecystitis and cholangitis, pancreatitis, gastritis, enteritis, duodenitis, oesophagitis |
| Haematologic irAE | Anaemia, thrombocytopenia, lymphopenia, neutropenia |
| Musculoskeletal irAE | Myositis alone or overlap manifestation (myositis and myocarditis and/or myasthenia gravis), inflammatory arthritis, arthralgias, myalgias, dermatomyositis, polymyalgia rheumatica or polymyalgia-like syndrome, Sjogren syndrome |
| Neurological irAE | Myasthenia gravis, peripheral neuropathy, Guillain-Barre syndrome, meningitis, encephalitis, myelitis, demyelinating disorders |
| Ocular irAE | Dry eyes, uveitis or iritis, myopathy, optic neuritis |
| Pulmonary irAE | Pneumonitis, interstitial lung disease |
| Renal irAE | Acute kidney injury, kidney failure, nephritis (interstitials, glomerular) |
| Skin irAE | Pruritus, rash (erythema, maculopapular and pustulopapular), dermatitis, vitiligo, erythema multiforme, eczematous, psoriasiform, toxic epidermal necrolysis |
|  |  |
|  |  |
|  |  |
